# ABCABC Stacking‐Enabled Non‐Centrosymmetry 3R‐ZnIn_2_S_4_ Nanosheets for Piezocatalytic Uranium Extraction

**DOI:** 10.1002/advs.76617

**Published:** 2026-07-16

**Authors:** Song Li, Huaijuan Zhou, Yingting Yang, Yanhong Lv, Zdeněk Sofer, Jianyun Zheng, Jinhua Li

**Affiliations:** ^1^ School of Materials Science and Engineering School of Interdisciplinary Science Beijing Institute of Technology Beijing China; ^2^ Beijing Key Laboratory of Intelligent Molecular Materials and High‐throughput Manufacturing School of Chemistry and Chemical Engineering Beijing Institute of Technology Beijing China; ^3^ School of Physics and Chemistry Hunan First Normal University Changsha China; ^4^ Department of Inorganic Chemistry University of Chemistry and Technology Prague Prague Czech Republic; ^5^ State Key Laboratory of Chem/Bio‐Sensing and Chemometrics, College of Chemistry and Chemical Engineering Hunan University Changsha China

**Keywords:** 3R‐ZnIn_2_S_4_, ABCABC stacking, H_2_O_2_ production, piezocatalysis, uranium extraction

## Abstract

Piezoelectric materials for uranium extraction have attracted increasing attention, as they offer a self‐powered, light‐independent strategy to harness ambient mechanical energy. However, natural mechanical energies are typically weak and low‐frequency, necessitating materials with high flexibility, facile deformation, and large surface area. Herein, we demonstrate that the ABCABC stacking sequence along the *c*‐axis in two‐dimensional (2D) 3R‐ zinc indium sulfide (3R‐ZnIn_2_S_4_) nanosheets induces intrinsic non‐centrosymmetry, enabling robust piezocatalysis for on‐site hydrogen peroxide (H_2_O_2_) generation and simultaneous uranium extraction. Density functional theory (DFT) calculations confirm that solvation effects enhance the lattice flexibility and intrinsic dipole moment of 3R‐ ZnIn_2_S_4_, resulting in the formation of an electron‐rich surface that thermodynamically favors the two‐electron oxygen reduction reaction (2e− ORR) for H_2_O_2_ generation. Under mild ultrasonic agitation (35 kHz, 50 W), the 3R‐ZnIn_2_S_4_ nanosheets achieved a H_2_O_2_ yield of 276.7 µmol g^−1^ h^−1^ and a uranium extraction capacity of 763.2 mg g^−1^. Mechanistic investigations elucidate that the uranium extraction process proceeds through a dual‐coordination pathway, integrating direct piezoelectric reduction and in situ H_2_O_2_‐mediated complexation of uranium species. Notably, 3R‐ZnIn_2_S_4_ demonstrates excellent cycling stability over five cycles, high selectivity toward uranium, and effective anti‐biofouling performance under ultrasonic conditions (35 kHz, 50 W). Collectively, our findings establish stacking‐induced piezoelectricity as a light‐independent strategy for sustainable uranium extraction to support nuclear energy development.

## Graphical abstract

The ABCABC stacking sequence in 2D ZnIn_2_S_4_ nanosheets creates intrinsic non‐centrosymmetry, enabling robust piezocatalysis under weak mechanical agitation. The nanosheets achieve a H_2_O_2_ yield of 276.7 µmol g^−1^ h^−1^ and uranium extraction capacity of 763.2 mg g^−1^ via a dual mechanism of direct piezoreduction and in situ H_2_O_2_ complexation.

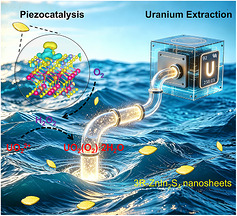

## 1 Introduction

According to the “World Nuclear Outlook Report (2026)” [1] and “World Nuclear Performance Report 2025” [2], the ongoing expansion of nuclear power infrastructure has placed existing uranium reserves under growing strain, highlighting the acute scarcity of terrestrial uranium resources. Extracting uranium from aqueous environments has emerged as a promising dual‐function strategy, not only to supplement uranium supply [3, 4] but also to remediate uranium‐contaminated wastewater arising from nuclear accidents and industrial effluents [5, 6, 7]. Uncontrolled release of uranium mine wastewater would introduce radioactive contaminants into aquatic ecosystems, posing severe ecological risks and potential threats to human health [8]. These challenges underscore that the development of efficient, eco‐friendly technologies for uranium extraction from water environments is pivotal to advancing the dual goals of energy sustainability and environmental protection [9, 10].

Water bodies possess abundant ambient mechanical energy in the form of fluid flow, vibration, waves, and other mechanical motions [11, 12]. These renewable, intermittent, and low‐frequency mechanical energies are omnipresent in flowing liquid environments, offering a perpetual, carbon‐neutral power source. To efficiently harness these low‐frequency mechanical energies, piezoelectric materials require high flexibility, facile deformation, and large surface area. Recent state‐of‐the‐art studies have enabled self‐powered uranium extraction by harvesting mechanical energy, a process termed piezocatalytic uranium extraction [13, 14, 15, 16]. For instance, ZnO/COF heterostructure [17] and BiFeO_3_@In_2_Se_3_ [14] have been successfully employed for this purpose. These landmark works established that the conversion of mechanical energy into chemical energy via piezocatalysis is a transformative strategy for self‐powered, sustainable uranium extraction. At the heart of this piezocatalytic technology lies the piezoelectric material, whose intrinsic physicochemical properties dictate the efficiency of mechanical‐to‐chemical energy conversion [18]. Yet conventional materials remain severely constrained by weak piezoelectric response, scarce active sites, and poor stability that collectively limit charge separation, uranium affinity, and operational longevity, thus driving an urgent need for rationally engineered alternatives.

2D layered materials have emerged as transformative piezocatalysts due to their ultrahigh surface area for uranyl (UO_2_^2+^) adsorption [19], mechanical flexibility for large deformation‐induced polarization, and tunable interlayer stacking for symmetry engineering [20, 21]. The efficacy of such 2D piezocatalysts is, however, fundamentally governed by crystal symmetry, as dictated by Neumann's principle; only non‐centrosymmetric structures exhibit piezoelectricity [22]. Atomic‐level control over symmetry is paramount for the rational design of advanced piezocatalysts. ZnIn_2_S_4_, a layered ternary chalcogenide with tunable electronic properties [20, 21], exemplifies this structure–property relationship by existing in two distinct polytypes: the 2H phase (hexagonal, *P6_3_/mc*) with ABAB stacking and the 3R phase (trigonal, *R3m*) with ABCABC stacking [23, 24, 25]. While both phases share nearly identical intralayer coordination, their centrosymmetry differs fundamentally due to their distinct stacking sequences. The 2H structure possesses a center of inversion [26, 27], rendering it centrosymmetric and thus piezoelectrically inactive in bulk form. To bypass this limitation, recent studies have resorted to defect engineering or gradient doping to break the local centrosymmetry of the 2H phase, achieving measurable piezoelectricity [28, 29, 30]. However, these extrinsic strategies typically introduce inhomogeneity and raise long‐term stability concerns. In contrast, although the non‐centrosymmetry of the 3R structure arising from its polar stacking along the *c*‐axis has been established [23, 24, 25], its intrinsic piezoelectricity for mechanical‐to‐chemical energy conversion remains underexplored [31].

Herein, we report that the ABCABC stacking along the *c*‐axis induces non‐centrosymmetry in 3R‐ZnIn_2_S_4_ nanosheets, which are synthesized via chemical vapor transport (CVT) and mechanical exfoliation, and demonstrate their exceptional performance for piezocatalytic uranium extraction. Unlike conventional hydrothermal synthesis, which typically yields flower‐like ZnIn_2_S_4_ assemblies composed of randomly oriented nanosheets [32, 33], the as‐synthesized 2D 3R‐ZnIn_2_S_4_ nanosheets are well‐defined and free‐standing, with large lateral dimensions and atomically clean surfaces. This anisotropic morphology not only preserves the intrinsic non‐centrosymmetric stacking feature of the 3R phase but also maximizes the exposure of mechanically responsive crystal facets, thereby facilitating more efficient strain transfer and polarization switching under mechanical agitation. Leveraging the unique piezoelectricity induced by ABCABC stacking, the 3R‐ZnIn_2_S_4_ nanosheets achieve a high uranium extraction capacity of 763.2 mg g^−1^ under mild, light‐independent mechanical agitation, along with excellent selectivity over competing metal ions, and remarkable antifouling performance in aqueous environments. Mechanistic studies reveal a dual‐coordination pathway for uranium extraction, which integrates direct piezoelectric reduction and in situ H_2_O_2_‐mediated complexation of uranium species. This work deepens the understanding of structure–property relationships in layered piezocatalysts and establishes a new paradigm for designing materials that synergistically harvest mechanical energy and recover strategic resources.

## 2 Results and Discussion

### 2.1 Stacking‐Induced Non‐Centrosymmetry and Piezoelectric Response

**FIGURE 1:**
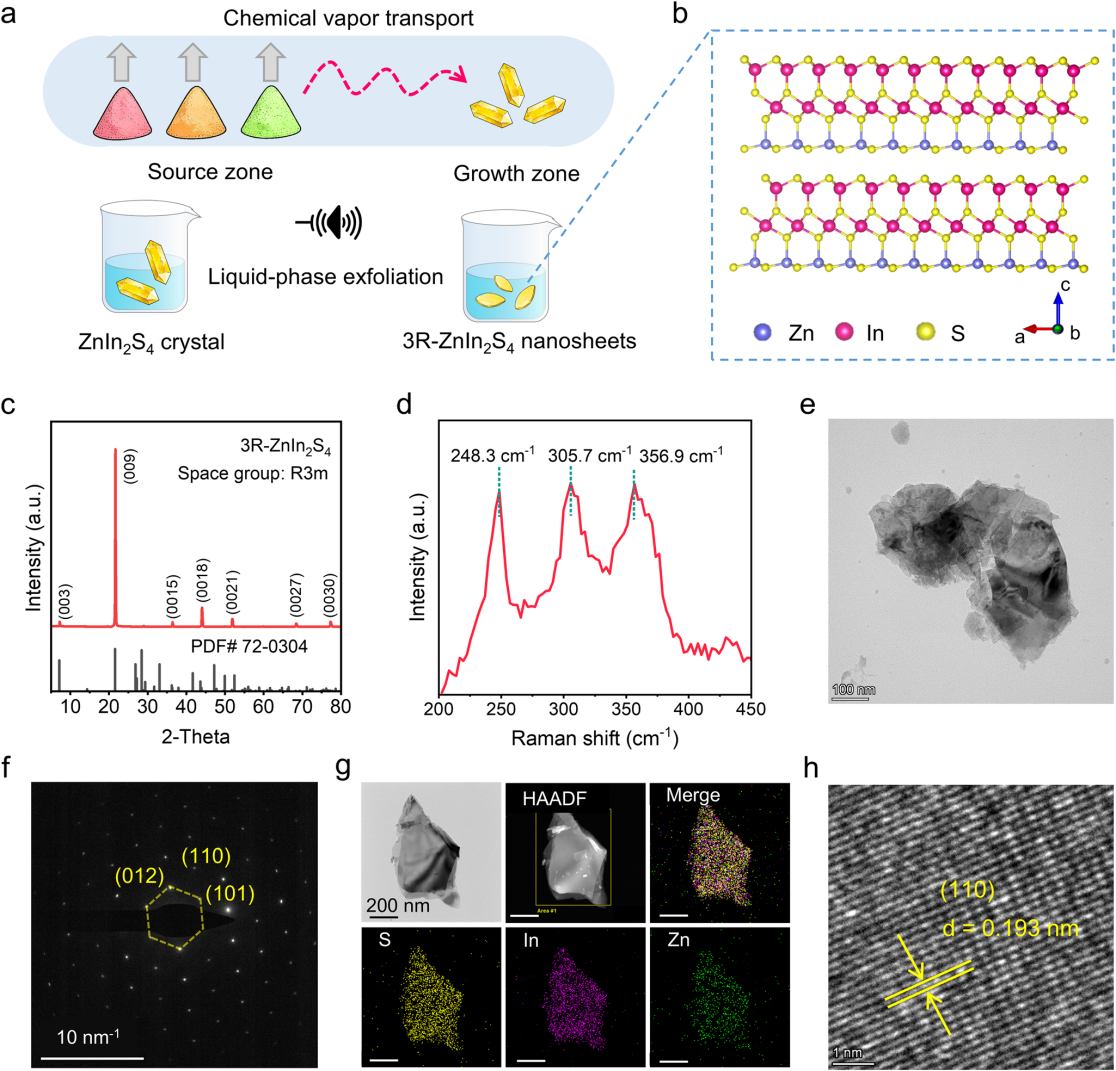
(a) Schematic illustration of the synthesis procedure. (b) Side view of the 3R‐ZnIn_2_S_4_ crystal structure. (c) XRD patterns and (d) Raman spectra of the as‐prepared 3R‐ZnIn_2_S_4_. (e) TEM image, (f) SAED pattern, (g) HAADF‐STEM image with corresponding elemental mappings (In, Zn, S), and (h) composite overlay. HRTEM image.

3R‐ZnIn_2_S_4_ nanosheets were fabricated via ultrasonic‐assisted liquid‐phase exfoliation of bulk ZnIn_2_S_4_ single crystals prepared by the CVT method, as illustrated in Figure 1a. Structurally, 3R‐ZnIn_2_S_4_ crystallizes in a hexagonal structure with an ABCABC stacking sequence along the c‐axis, wherein layers of identical orientation undergo relative dislocation (Figure 1b and Figure S1). This unique stacking mode is critical as it can break inversion symmetry from the monolayer to the bulk [24], thereby endowing 3R‐ZnIn_2_S_4_ (belonging to the non‐centrosymmetric *R3m* (160) space group) with intrinsic piezoelectricity [34]. The phase purity and crystallinity of the as‐prepared 3R‐ZnIn_2_S_4_ were systematically verified by complementary characterizations. As shown in Figure 1c, X‐ray diffraction (XRD) analysis confirmed that the synthesized ZnIn_2_S_4_ crystallizes in the 3R phase (PDF#72‐0304). The diffraction peaks at 7.1°, 21.5°, 36.4°, 44.2°, 51.9°, 68.4°, and 77.3° correspond to the (003), (009), (0015), (0018), (0021), (0027), and (0030) crystal planes, respectively. The exclusive presence of (00l)‐type diffraction peaks indicates a strong preferential orientation along the c‐axis, which confirms the controlled growth direction of the 2D nanosheets. This highly oriented texture is essential for maximizing the net piezoelectric response along the polar axis. Raman spectroscopy further validated the phase assignment of the 3R‐ZnIn_2_S_4_. As presented in Figure 1d, the Raman spectrum exhibits three characteristic phonon modes in the range of 200–450 cm^−1^, which match well with the expected vibrational features of the *R3m* (160) space group symmetry for 3R‐ZnIn_2_S_4_ [35]. Specifically, the peak at 248.3 cm^−1^ corresponds to the out‐of‐plane A_1g_ mode along the c‐axis, while the peaks at 305.7 and 356.9 cm^−1^ are associated with the in‐plane E_g_ modes within the ab plane. The coexistence of out‐of‐plane (A_1g_) and in‐plane (E_g_) vibrational modes further confirms the inherent anisotropic structural characteristics inherent to the non‐centrosymmetric 3R phase.

The 2D ultrathin morphology and layered structure of 3R‐ZnIn_2_S_4_ were characterized by transmission electron microscopy (TEM) and high‐resolution TEM (HRTEM). Figure 1e shows a typical 2D nanosheet structure, with a measured hydrodynamic diameter of ∼360 nm (Figure S2a) and a zeta potential ranging from −2.6 to −4.4 mV (Figure S2b). This atomically thin geometry not only maximizes the exposed surface area but also facilitates mechanical deformation under external stress, both of which are favorable for piezocatalytic reactions. The selected‐area electron diffraction (SAED) pattern in Figure 1f exhibits sharp periodic diffraction spots, which is consistent with the trigonal phase of 3R‐ZnIn_2_S_4_ and reflect a high crystallinity of the nanosheets. Notably, the spotty, rather than ring‐like pattern, confirms the single‐crystalline nature of the as‐exfoliated nanosheets. High‐angle annular dark‐field STEM (HAADF‐STEM) imaging and corresponding energy‐dispersive X‐ray spectroscopy (EDS) elemental mapping (Figure 1g) further confirm the homogeneous distribution of In, Zn, and S across the entire nanosheet, indicating high phase purity and compositional uniformity. Quantitative TEM‐EDX analysis of the 3R‐ZnIn_2_S_4_ nanosheets (Figure S3) yields an atomic composition of Zn (13.19%), In (27.65%), and S (59.16%), which closely matches the theoretical stoichiometry of 1:2:4. Further structural details are revealed by the HRTEM image in Figure 1h, which exhibits distinct lattice fringes with an interplanar spacing of 0.193 nm, corresponding to the (110) crystal planes. Compared to previously reported ZnIn_2_S_4_ samples synthesized via hydrothermal methods [36, 37], which commonly assemble into flower‐like hierarchical architectures consisting of randomly oriented and entangled nanosheets, our CVT strategy enables the fabrication of well‐defined, free‐standing 2D nanosheets with clean surfaces and preserved crystallographic orientation.

**FIGURE 2:**
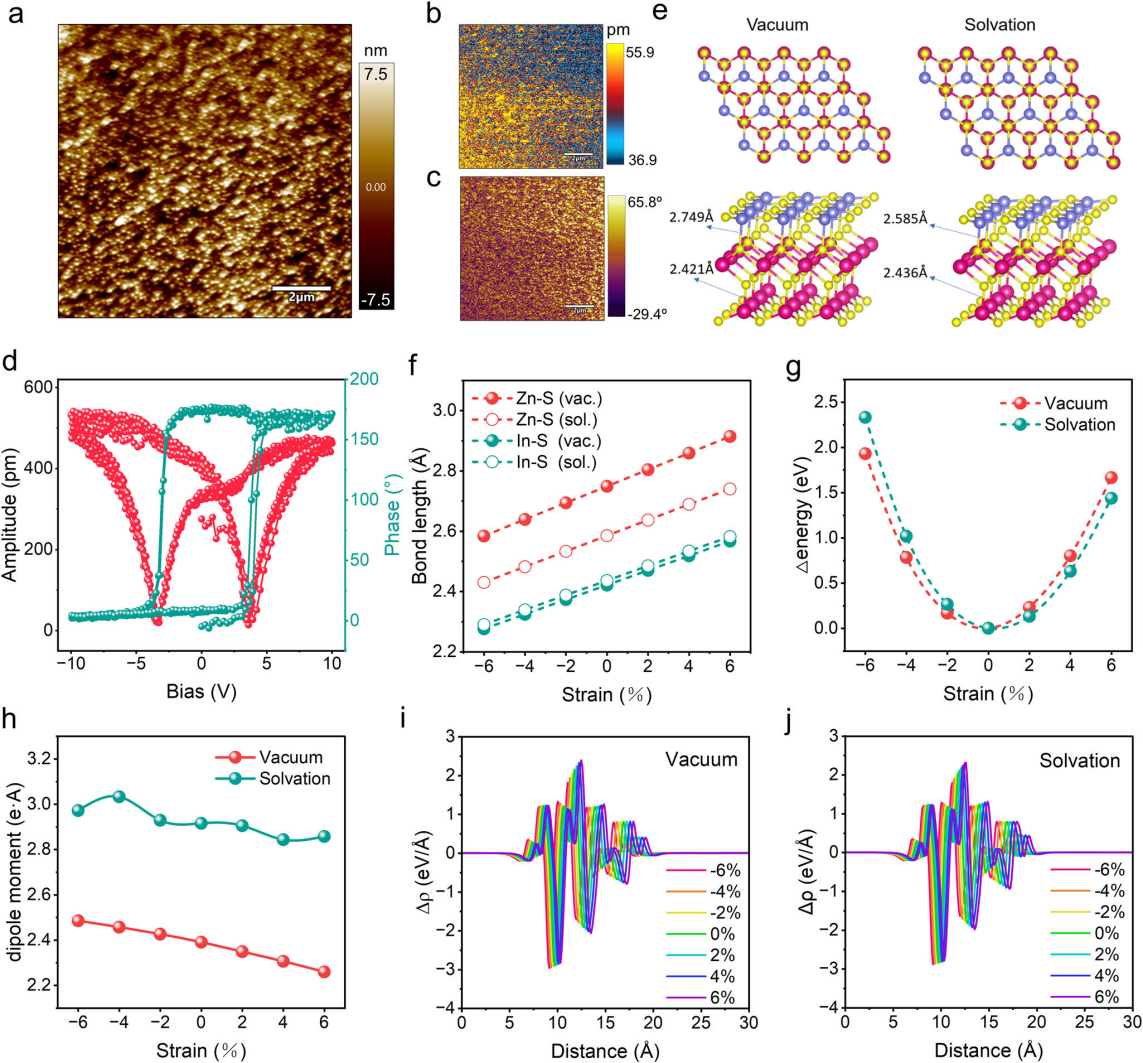
(a) Topographic image of 3R‐ZnIn_2_S_4_ nanosheets. (b) Out‐of‐plane phase image. (c) Out‐of‐plane piezoelectric amplitude response. (d) Typical amplitude–voltage butterfly loop curves. (e) Comparison of S─Zn and S─In bond lengths under vacuum and solvation. (f) Changes in S─Zn and S─In bond lengths as a function of uniaxial strain under vacuum and solution conditions. (g) Change in total energy (ΔE) as a function of uniaxial strain along the *c*‐axis under vacuum and solution conditions. (h) Change in Δ*ρ* as a function of uniaxial strain under vacuum and solution conditions. Spatial distribution of the strain‐induced potential gradient under (i) vacuum and (j) solvation.

To directly verify the piezoelectricity derived from the ABCABC stacking sequence, we performed piezoresponse force microscopy (PFM) measurements on the as‐exfoliated 3R‐ZnIn_2_S_4_ nanosheets. The topographic image (Figure 2a) reveals a well‐defined flake‐like nanostructure. Both the out‐of‐plane phase image and the piezoelectric amplitude response exhibit distinct domain features (Figure 2b,c), with the appreciable piezoelectric displacement amplitude providing direct evidence of a pronounced local piezoelectric effect. Furthermore, upon applying a bias ranging from −10 to +10 V, localized piezoelectric displacement was induced, accompanied by ∼180° domain switching. The corresponding displacement–voltage curve displays a characteristic butterfly hysteresis loop (Figure 2d). From this butterfly loop, an effective piezoelectric coefficient d_33_ of 65 pm/V is derived based on the linear fit of the displacement–voltage slope (Figure S4). Collectively, these results confirm that the non‐centrosymmetry induced by the ABCABC stacking sequence endows 3R‐ZnIn_2_S_4_ with robust piezoelectricity, laying a solid structural foundation for its application as a piezocatalyst in aqueous environments.

Given that the mechanical and polarization properties of 3R‐ZnIn_2_S_4_ are pivotal to its piezocatalytic performance in uranium extraction, DFT calculations with implicit solvation were carried out to systematically compare its structural and electronic behavior under vacuum and aqueous solution conditions. As shown in Figure 2e, solvation induces opposite bond length changes: the S─Zn bond is shortened, whereas the S─In bond is elongated. This divergence originates from the distinct electronic structures of Zn^2+^ and In^3+^ ions. The smaller Zn^2+^ with a higher‐charge‐density undergoes stronger dielectric screening, which reduces the ionic character of the Zn─S bond and promotes increased orbital overlap, thereby leading to bond contraction. In contrast, the larger In^3+^ ion with more delocalized electron density experiences a classical screening effect that weakens electrostatic attraction and elongates the S─In bond. This solvation‐induced differential bond length variation suggests that the lattice undergoes a complex yet reversible reorganization in aqueous media. Such reorganization may facilitate efficient strain transfer within the material and enhance its piezoelectric polarization under mechanical agitation. To further quantify the effect of solvation on the material's mechanical response of 3R‐ZnIn_2_S_4_ beyond equilibrium bond lengths, uniaxial strain ranging from –6% to 6% was applied along the *c*‐axis (Figures S5 and S6). Under this strain regime, both S─Zn and S─In bond lengths exhibit a linear dependence on the applied strain (Figure 2f). Importantly, despite the stress‐induced bond length changes, the deformation remains elastic, meaning the lattice can fully recover its original structure upon removal of the external without sustaining permanent damage.

Figure 2g presents the total energy change (Δ*E*) as a function of uniaxial strain along the *c*‐axis under both vacuum and solution conditions. The results reveal an asymmetric solvation effect that is dependent on the strain direction. In the compressive strain regime (from −6% to 0%), the deformation energy in solution is lower than that in vacuum, indicating that the lattice becomes softer under compression in the aqueous environment [38]. Conversely, in the tensile strain regime (from 0% to +6%), the deformation energy in solution is higher than that in vacuum, suggesting that the lattice becomes stiffer under tension when solvated. As summarized in Table S1, although the material stiffens under tension, the magnitude of softening under –6% compressive strain (Δ*E* reduction ≈ 0.4017 eV) is substantially larger than the magnitude of stiffening under +6% tension (Δ*E* increase ≈ 0.2273 eV). Therefore, under typical mechanical agitation involving alternating compressive and tensile cycles, the net effect is an overall enhancement in mechanical compliance. This net softening implies that the material exhibits greater equilibrium compliance, which may potentially enhance its piezoelectric polarization and piezocatalytic performance for uranium extraction in an aqueous environment.

Building on the observed softening effect, the implications for piezoelectric polarization were further investigated. The electron density distribution along the *Z*‐direction and the corresponding integrated dipole moment were calculated under both vacuum and solvation conditions (Figure S7). To elucidate the effect of solvation on polarization, the strain‐dependent variation in dipole moment was analyzed. As shown in Figure 2h, the polarization response exhibits a uniform enhancement under aqueous conditions. Specifically, the dipole moment shows consistently higher absolute values across the entire strain range, while its strain dependence remains comparable to that under vacuum conditions. These results indicate that the aqueous environment counteracts the depolarization effect, leading to a significant enhancement in absolute polarization without altering the intrinsic strain sensitivity of 3R‐ZnIn_2_S_4_. To further clarify how solvation modulates the piezoelectric response under realistic working conditions, the spatial distribution of the potential gradient (Δ*ρ*, a key descriptor of the built‐in electric field) was investigated under varying strain levels. Under vacuum conditions, the piezoelectric field is confined to ∼5–20 Å, decaying to zero beyond 20 Å, which confirms strictly interfacial charge redistribution (Figure 2i,j). Compressive strains generate a negative potential gradient, while tensile strains generate a positive field, demonstrating bidirectional strain‐tunable piezoelectricity. A residual field persists at 0% strain due to surface charge reconstruction. Under solvation conditions, Δ*ρ* is slightly reduced across all strains due to partial dielectric screening; however, the strain‐dependent modulation of the electric field remains robust, and the bidirectional piezoelectric response is preserved. This ensures that mechanical vibration can still generate a measurable piezoelectric response.

### 2.2 Thermodynamically Favorable H_2_O_2_ Evolution

**FIGURE 3:**
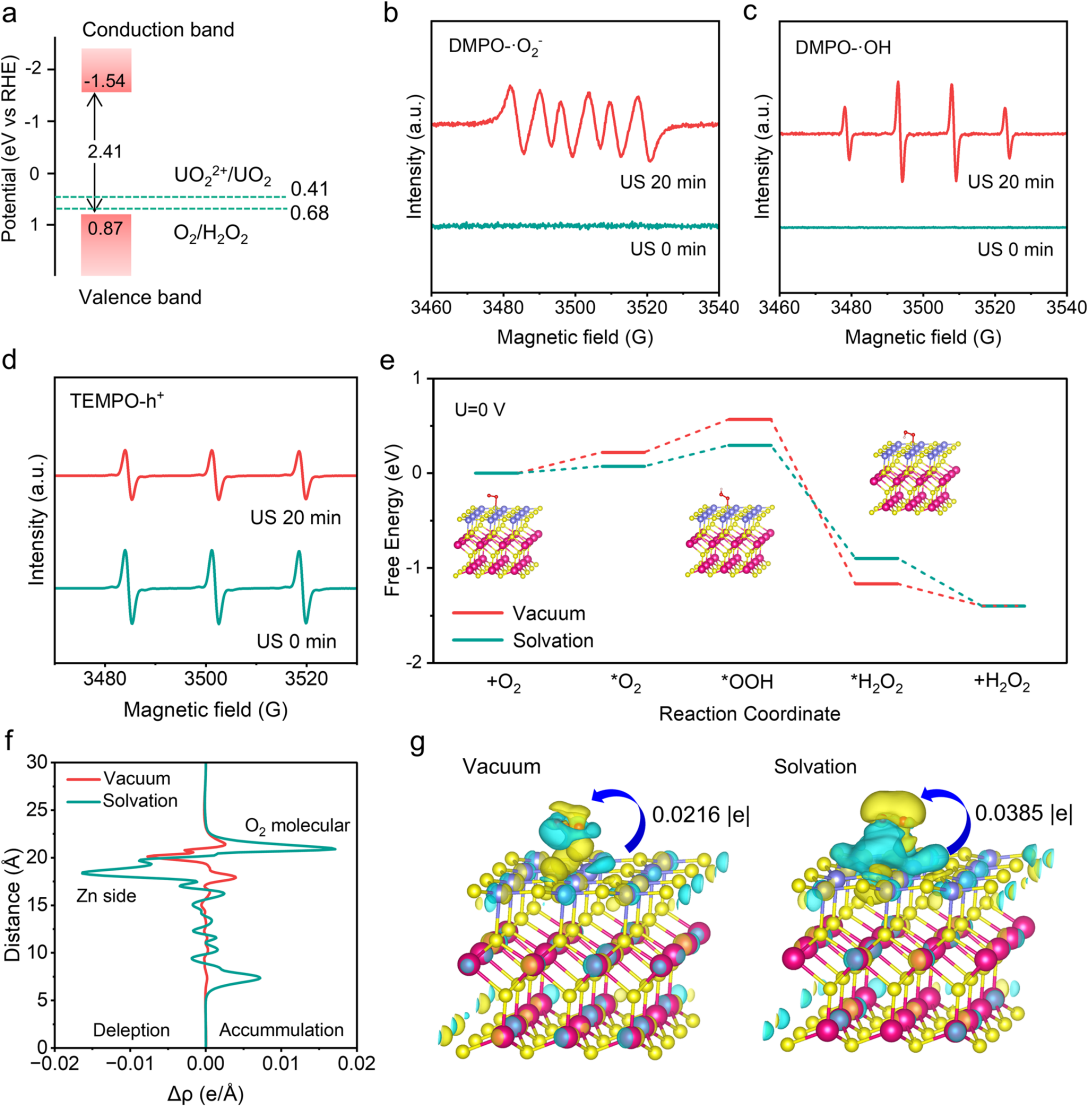
(a) Energy band diagram determined from UV–vis DRS, Mott–Schottky plots and VB‐XPS results. EPR signals of DMPO‐trapped (b) •O_2_^−^ and (c) •OH radicals under ultrasonic irradiation. (d) EPR spectra with TEMPO as the hole scavenger. (e) Gibbs free energy profiles of the ‐2e− ORR pathway toward H_2_O_2_ formation in vacuum and implicit solvation environments. (f) Charge density difference for O_2_ adsorbed on the (110) surface of 3R‐ZnIn_2_S_4_ at 0% strain (yellow: charge accumulation; blue: charge depletion). (g) Bader charge analysis of adsorbed O_2_ in vacuum and solvation conditions.

Typically, H_2_O_2_ generation serves as a key step in mediating uranium extraction [39, 40]. Based on the established enhanced piezoelectric polarization of 3R‐ZnIn_2_S_4_ under solvation, we next examined its electronic band structure to assess its thermodynamic feasibility for piezocatalytic H_2_O_2_ production. The bandgap of 3R‐ZnIn_2_S_4_ was analyzed by ultraviolet‐visible diffuse reflectance spectroscopy (UV‐vis DRS). As shown in Figure S8, 3R‐ZnIn_2_S_4_ exhibits broad optical absorption across the visible spectrum, with an optical bandgap energy (*E*_g_) determined to be approximately 2.41 eV. Mott–Schottky (M–S) measurements (Figure S9) confirm n‐type semiconductor behavior, with a flat‐band potential (*E*_fb_) of −0.23 V versus reversible hydrogen electrode (RHE). Valence band X‐ray photoelectron spectroscopy (VB‐XPS) in Figure S10 places the valence band maximum (VBM) at 0.87 eV vs. RHE, from which the conduction band minimum (CBM) is calculated to be −1.54 eV vs. RHE using the relation *E*_CB_ = *E*_VB_—*E*_g_. The resulting energy band diagram is presented in Figure 3a. The positions of the CBM and VBM are well‐aligned with the thermodynamic potentials required for the two‐electron ORR to H_2_O_2_ [41], confirming that 3R‐ZnIn_2_S_4_ is a promising candidate for piezocatalytic H_2_O_2_ production.

To experimentally verify the ROS‐mediated ORR pathway predicted by the band structure, electron paramagnetic resonance (EPR) trapping experiments were conducted using 5,5‐dimethyl‐1‐pyrroline‐N‐oxide (DMPO) as the spin‐trapping agent. Characteristic signals assigned to DMPO–•O_2_^−^ (superoxide radical) and DMPO–•OH (hydroxyl radical) adducts emerged rapidly within 20 min of ultrasonic irradiation (Figure 3b,c). The simultaneous detection of •O_2_^−^ and •OH not only confirms a 2e− ORR mechanism but also validates the thermodynamic feasibility of ROS‐mediated H_2_O_2_ evolution. Specifically, the superoxide radical (•O_2_^−^) originates from the one‐electron ORR (E (•O_2_^−^/O_2_) = −0.33 V versus reversible hydrogen electrode (NHE)), while the •OH arises primarily from the subsequent decomposition of H_2_O_2_ rather than from direct water oxidation by holes, as the latter pathway is thermodynamically less favorable (E(H_2_O/•OH) = +2.73 V vs. NHE) [42]. To further clarify the contribution of photogenerated holes in the piezocatalytic process, 2,2,6,6‐tetramethylpiperidinyloxy (TEMPO) was utilized as a hole scavenger. A distinct attenuation of the characteristic signal was observed under ultrasonic excitation (Figure 3d), confirming the efficient consumption of piezoelectrically induced holes. This hole consumption, rather than significantly contributing to •OH production via thermodynamically unfavorable water oxidation, suppresses electron–hole recombination and thereby enriches surface electrons available to drive the 2e− ORR toward H_2_O_2_ synthesis. To further corroborate the piezocatalytic potential for H_2_O_2_ production, transient current measurements and electrochemical impedance spectroscopy (EIS) were performed. As detailed in Figures S11 and S12, 3R‐ZnIn_2_S_4_ exhibits a detectable piezoelectric current under ultrasonic mechanical excitation, while EIS reveals a reduced charge‐transfer resistance with increasing applied bias voltage. Collectively, these results confirm that 3R‐ZnIn_2_S_4_ thermodynamically enables favorable H_2_O_2_ evolution via piezocatalysis.

To gain atomic‐level insight into the experimentally observed H_2_O_2_ evolution, DFT calculations were performed on the 3R‐ZnIn_2_S_4_ surface. The Gibbs free energy changes (ΔG) for the 2e− ORR pathway (O_2_ + 2H^+^ + 2e^−^ → H_2_O_2_) [43] are presented in Figure 3e. The rate‐determining step (^*^OOH → H_2_O_2_) exhibits a low overpotential under 0% strain [44]. To better reflect the actual catalytic environment, the reaction intermediates were further optimized under implicit solvation conditions (Figure S13). The solvation environment stabilizes the key ^*^OOH intermediate through hydrogen bonding interactions, resulting in a more favorable adsorption configuration compared to that under vacuum. Consequently, as shown in Figure 3e, the overall reaction free energy under solvation is significantly lower than that in vacuum, indicating that the aqueous environment further enhances the thermodynamic feasibility of H_2_O_2_ evolution. The charge density difference was further analyzed to visualize the interfacial charge redistribution upon O_2_ adsorption on the (001) surface of 3R‐ZnIn_2_S_4_ under 0% strain (Figure 3f). Under vacuum, charge transfer occurs from the adjacent Zn site to the adsorbed O_2_ molecule, with Zn acting as the electron donor. The charge transfer pattern shows charge accumulation (yellow isosurfaces) primarily on the adsorbed O_2_ and adjacent Zn sites, and charge depletion (blue isosurfaces) on the surrounding S atoms. This distinct pattern indicates strong electronic coupling between O_2_ and the catalyst surface, facilitating its activation toward the ^*^OOH intermediate. Under solvation conditions, the intrinsic piezoelectric polarization field of 3R‐ZnIn_2_S_4_ further enhances electron donation from Zn to O_2_, significantly promoting the 2e− ORR. This solvation‐enhanced charge transfer is quantitatively confirmed by Bader charge analysis (Figure 3g), which reveals that the amount of charge transferred from the 3R‐ZnIn_2_S_4_ surface to the adsorbed O_2_ increases from 0.0216 |e| (in vacuum) to 0.0385 |e| (under solvation). Collectively, these results demonstrate that the intrinsic piezoelectricity of 3R‐ZnIn_2_S_4_, particularly under aqueous conditions, enables both thermodynamically favorable and electronically facilitated H_2_O_2_ evolution.

### 2.3 On‐Site H_2_O_2_ Generation for Uranium Extraction

Building upon the confirmed thermodynamically favorable piezocatalytic H_2_O_2_ generation over 3R‐ZnIn_2_S_4_, we further explored its practical application potential for uranium extraction from uranium mine wastewater. As evidenced by the time‐dependent absorption spectra in Figure S14, a distinct absorption peak at 351 nm emerged and progressively intensified under ultrasonication in pure water under ambient air conditions. This monotonic increase in absorbance unambiguously confirms the stable and continuous piezocatalytic generation of H_2_O_2_. Quantitatively, the H_2_O_2_ yield rates reached 276.7 µmol g^−1^ h^−1^ in pure water, with first‐order rate constants (K_f_) of 1.75 µm min^−1^. Notably, benefiting from the high crystallinity of the as‐synthezised 2D nanosheets, the 3R‐ZnIn_2_S_4_ exhibited exceptional structural and catalytic stability. As presented in Figure S15, the H_2_O_2_ production yield remained nearly unchanged over five consecutive cycling tests, demonstrating robust reusability and long‐term durability for continuous piezocatalytic operation under mechanical vibration. To unambiguously identify the dominant reactive species and clarify the H_2_O_2_ formation pathway, systematic radical quenching experiments were conducted (Figure S16). First, a control experiment was performed by purging the reaction system with Ar for 30 min to remove dissolved oxygen. Under this condition, the H_2_O_2_ yield was dramatically suppressed, unequivocally confirming that the 2e−ORR is the dominant pathway for H_2_O_2_ generation. The introduction of *p*‐benzoquinone (a •O_2_^−^ scavenger) induced a dramatic decline in H_2_O_2_ output, firmly confirming that •O_2_^−^ serves as the pivotal intermediate governing H_2_O_2_ generation via sequential reduction and disproportionation. Meanwhile, triethanolamine (a typical hole scavenger) slightly promoted H_2_O_2_ production, suggesting that the consumption of piezoelectrically excited holes suppresses undesirable electron–hole recombination and enriches surface free electrons to facilitate oxygen reduction. In contrast, the introduction of *tert*‐butanol (TBA, a •OH scavenger) and sodium bromate (NaBrO_3_, an electron trapping agent) both reduced H_2_O_2_ evolution, revealing that piezoelectrically excited electrons and hydroxyl radicals also participate in the overall catalytic reaction network. NaBrO_3_ acts as an electron trap, capturing piezoelectrically excited electrons and thereby inhibiting the electron‐driven ORR pathway, which suppresses H_2_O_2_ generation. Separately, TBA scavenges •OH radicals. While •OH primarily originates from the decomposition of H_2_O_2_, its elimination may disturb secondary oxidative reactions that positively influence the net H_2_O_2_ yield, resulting in decreased H_2_O_2_ evolution. Mechanistically, these results confirm that H_2_O_2_ production is dominated by the selective 2e^−^ ORR, with negligible contribution from the competing four‐ectron pathway to H_2_O. This assignment is further substantiated by rotating disk electrode (RDE) measurements (Figure S17). At applied potentials of −0.4, −0.5, and −0.6 V, the calculated electron transfer numbers (n) are 1.88, 2.05, and 2.25, respectively, yielding a mean value of 2.05 ± 0.18. These values consistently fall close to 2, conclusively demonstrating that the 2e^−^ ORR pathway prevails on 3R‐ZnIn_2_S_4_. Consequently, the built‐in piezoelectric field of 3R‐ZnIn_2_S_4_ enables sufficient charge separation and suppresses electron–hole recombination, creating an electron‐rich catalyst surface ideally suited for selective 2e^−^ ORR.

**FIGURE 4:**
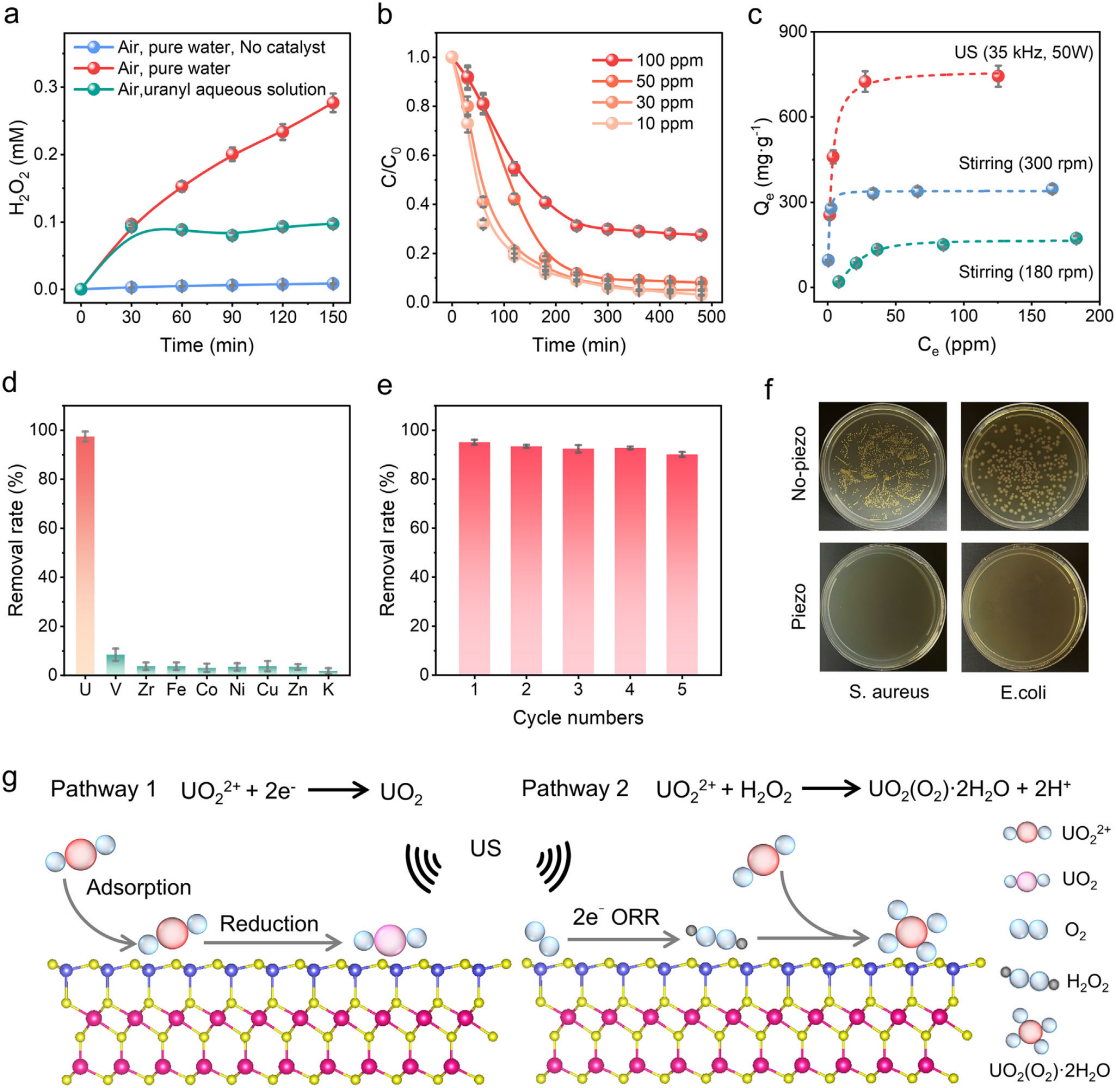
(a) H_2_O_2_ generation under different conditions: without 3R‐ZnIn_2_S_4_ (pure water), with 3R‐ZnIn_2_S_4_ alone, and with 3R‐ZnIn_2_S_4_ in the presence of 50 ppm UO_2_^2+^. (b) Piezocatalytic uranium extraction efficiencies at initial UO_2_^2+^ concentrations of 10, 30, 50, and 100 ppm. (c) Comparison of uranium uptake capacities of 3R‐ZnIn_2_S_4_ under different mechanical energy input conditions. (d) Ion selectivity measurement in the presence of competing metal ions, including V^5+^, Zr^4+^, Fe^3+^, Co^2+^, Ni^2+^, Cu^2+^, Zn^2+^, and K^+^. (e) Recyclability of 3R‐ZnIn_2_S_4_ for U(VI) extraction over five cycles. (f) Spread plate images showing anti‐biofouling performance against *S. aureus* and *E. coli*. (g) Schematic illustration of the dual‐pathway synergistic piezocatalytic uranium immobilization mechanism on 3R‐ZnIn_2_S_4_.

With piezocatalytic H_2_O_2_ generation firmly established, we next evaluated its application for uranium extraction. As shown in Figure 4a, a control experiment confirmed that under identical ultrasonic conditions but without 3R‐ZnIn_2_S_4_, only an extremely small amount of H_2_O_2_ was generated, indicating the piezocatalyst is responsible for the dominant H_2_O_2_ production. In the presence of 50 ppm uranium, the detected H_2_O_2_ concentration was consistently lower than that in uranium‐free environments, with a pronounced difference emerging after 30 min of reaction. This observation indicates that the in situ generated H_2_O_2_ is continuously consumed by reaction with UO_2_^2+^ to form uranium peroxide precipitates, rather than accumulating freely in solution. As the reaction proceeded, H_2_O_2_ generated via the 2e^−^ ORR pathway triggered the subsequent precipitation reaction with UO_2_^2+^, leading to the formation of (UO_2_)O_2_·2H_2_O [40]. Consequently, the H_2_O_2_ concentration ceased its continuous increase, reflecting its effective consumption in the uranium precipitation process. To further evaluate the piezocatalytic performance of the in situ H_2_O_2_ generation system for uranium extraction, systematic experiments were performed in U(VI) solutions with concentrations ranging from 10 to 100 ppm (Figure 4b). The piezocatalytically driven uranium extraction efficiencies reached 97%, 95%, 92%, and 72.5% for 10, 30, 50, and 100 ppm U(VI), respectively, demonstrating high extraction efficiency across a wide concentration range. Subsequent adsorption kinetic experiments further confirmed the critical role of piezocatalysis in enhancing uranium extraction (Figure 4c). Notably, under ultrasonic‐induced piezoelectric activation (35 kHz, 50 W), the maximum uranium adsorption capacity of 3R‐ZnIn_2_S_4_ reached 763.2 mg g^−1^. Furthermore, under milder mechanical energy inputs that better simulate practical fluid environments, mechanical stirring at 180 rpm and magnetic stirring at 300 rpm, the material achieved uranium uptake capacities of 177.3and 346.8 mg g^−1^, respectively, confirming that even weak mechanical shear forces can generate sufficient piezoelectric charge density to promote uranium immobilization. Time‐dependent adsorption studies revealed that uranium uptake increased rapidly within the initial 120 min, followed by a gradual slowdown, and reached adsorption equilibrium within 300 min. Detailed kinetic fitting (Figure S18) confirmed a combination of physical adsorption and chemisorption‐dominated mechanism mediated by the piezocatalytic reaction.

In practical uranium extraction processes from uranium mine wastewater, the complex ionic matrix inherent to such aqueous environments imposes stringent requirements on the ion selectivity of the employed 2D piezocatalysts. To evaluate the anti‐interference capability of 3R‐ZnIn_2_S_4_, U(VI) extraction experiments were conducted in the presence of all the following competing metal ions coexisting in the same solution, including V^5+^, Zr^4+^, Fe^3+^, Co^2+^, Ni^2+^, Cu^2+^, Zn^2+^, and K^+^, each at 50 ppm. As illustrated in Figure 4d, the 3R‐ZnIn_2_S_4_ maintained a significantly higher U(VI) extraction efficiency compared to its counterpart, demonstrating its superior selectivity and robustness in complex aqueous environments. The outstanding U(VI) selectivity originates from an H_2_O_2_‐mediated reaction, which selectively reacts with uranyl ions to form an insoluble uranyl peroxide precipitate, while none of the competing ions form stable or isolable peroxide solids under identical conditions. In addition to the selective precipitation mechanism, solution pH plays a critical role in the uranium extraction performance. Real uranium mine wastewater typically exhibits a weakly acidic to near‐neutral pH (4.0–7.0) range due to the presence of residual sulfuric acid or carbonates from ore processing. As shown in Figure S19, experiments conducted over the pH range of 3–11 demonstrate that 3R‐ZnIn_2_S_4_ achieves optimal U(VI) removal efficiency (>90%) between pH 5 and 7. In this pH window, uranyl ions predominantly exist as UO_2_^2+^ and its hydrolyzed species (e.g., (UO_2_)_2_(OH)_2_^2+^), which readily react with piezocatalytically generated H_2_O_2_ to form an insoluble uranyl peroxide precipitate. At pH < 3, proton competition and the high solubility of uranyl species suppress precipitation; at pH > 9, the formation of anionic uranyl‐carbonate complexes (e.g., UO_2_(CO_3_)_3_^4−^) inhibits the interaction with H_2_O_2_. Furthermore, 3R‐ZnIn_2_S_4_ retained high piezocatalytic efficiency over five cycles with no significant activity loss (Figure 4e), confirming its stability and reusability. Aquatic environments also contain a variety of microorganisms that tend to accumulate on material surfaces to form dense biofilms, which block active sites, impair catalytic activity, and even damage the material structure, leading to performance degradation. We further evaluated the antifouling performance of 3R‐ZnIn_2_S_4_ nanosheets. Spread plate images before and after antibacterial treatment are shown in Figure S20 and Figure 4f. Following 120 min of ultrasonication, the material demonstrated strong antibacterial efficacy, inactivating nearly 100% of both *S. aureus* and *E. coli*. This effect is attributed to the physical disruption of bacterial membranes by the sharp edges of the nanosheets [45], combined with the ultrasonication‐induced generation of •O_2_^−^ and •OH [46]. To distinguish between these two contributions, a control experiment was performed under identical ultrasonic conditions but without 3R‐ZnIn_2_S_4_. Under this control condition, only an extremely small amount of bacterial inactivation was observed, indicating that the mechanical effect of ultrasound alone is negligible. Therefore, the dominant antibacterial mechanism is attributed to piezocatalytically generated ROS. Such inherent anti‐biofouling capability effectively inhibits biofilm formation, thereby ensuring sustained extraction performance in biologically active environments.

Based on the aforementioned experimental and computational results, a dual‐pathway synergistic piezocatalytic uranium immobilization mechanism on 3R‐ZnIn_2_S_4_ is proposed [40], as illustrated in Figure 4g. The first pathway involves direct piezoelectric reduction: surface electrons enriched by the piezoelectric field directly reduce soluble UO_2_^2+^ to insoluble UO_2_. The second pathway involves in situ H_2_O_2_‐mediated complexation, where the piezoelectric field drives the 2e^−^ ORR to generate H_2_O_2_, which subsequently reacts with UO_2_^2+^ to form stable uranyl peroxide hydrate precipitates (UO_2_(O_2_)·2H_2_O), as confirmed by subsequent analyses. Crucially, these two pathways are not independent but chemically coupled. The initially formed U(IV) can be further oxidized by H_2_O_2_ to yield the same uranyl peroxide product. This coupling transforms a fraction of the direct reduction product into the complexation product, demonstrating that the two routes cooperate rather than compete. Mechanistically, the direct reduction pathway consumes surface electrons, suppressing charge recombination and thereby enhancing the overall piezocatalytic efficiency. Concurrently, the H_2_O_2_‐mediated pathway provides a highly selective and stable secondary immobilization route.Together, they establish a robust, dual‐mode uranium fixation system. The detailed reaction steps and key intermediate species are summarized in Table S2.

### 2.4 Piezo‐Extracted Products Analysis

**FIGURE 5:**
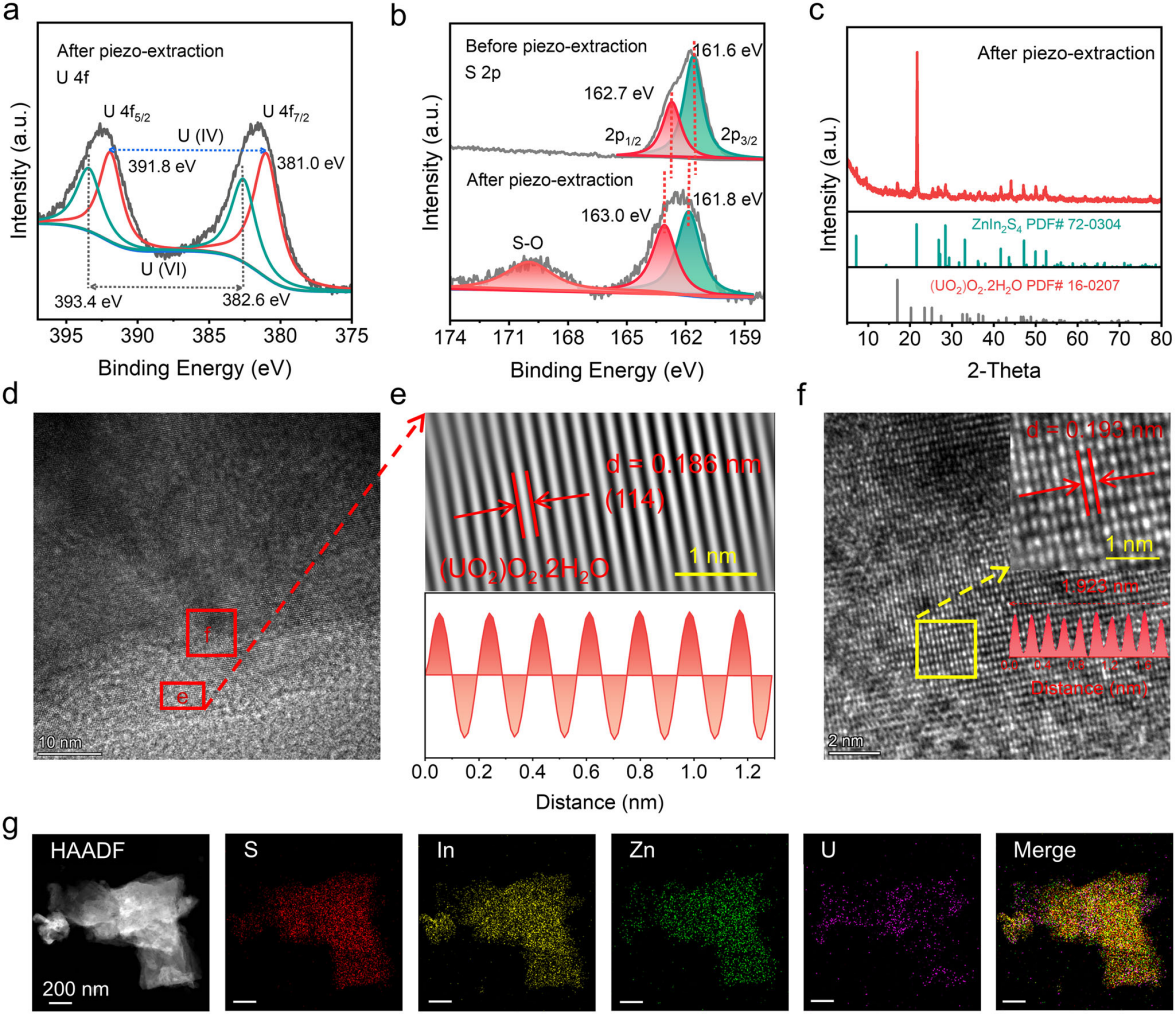
Comprehensive characterization of piezocatalytically extracted uranium species using 3R‐ZnIn_2_S_4_. (a) high‐resolution U 4f XPS spectra showing coexistence of U(IV) and U(VI); (b) S 2p XPS spectra demonstrating binding energy shifts and S─O bond formation after extraction; (c) XRD patterns confirming UO_2_ and UO_2_(O_2_)·2H_2_O phases; (d) TEM image revealing nanosheet morphology; (e, f) HRTEM images with lattice spacings corresponding to UO_2_(O_2_)·2H_2_O (114) and 3R‐ZnIn_2_S_4_ (110) planes; (g) EDX elemental mapping illustrating uniform distribution of S, Zn, In, and U.

To further clarify the phase evolution of uranium species during the dual‐pathway piezocatalytic process proposed above, the piezo‐extracted products derived from 3R‐ZnIn_2_S_4_ were further subjected to comprehensive characterization via XPS, XRD, and HRTEM. The survey XPS spectrum clearly reveals an obvious uranium signal after piezoelectric uranium extraction by 3R‐ZnIn_2_S_4_ (Figure S19a). As depicted in the high‐resolution U 4f XPS spectra (Figure 5a), prominent characteristic peaks centered at 391.8 and 381.0 eV are unambiguously assigned to the U 4f_5/2_ and U 4f_7/2_ orbitals of U(IV), respectively. Meanwhile, additional distinct peaks at 393.4 and 382.6 eV corresponding to the typical binding energy features of U(VI). These observations confirm the coexistence of U(IV) and U(VI) in the final products, which is highly consistent with the proposed dual‐pathway mechanism: U(VI) is partially reduced to U(IV) via direct piezoelectric reduction (Pathway 1) and partially converted to U(VI)‐containing uranyl peroxide precipitates through in situ H_2_O_2_‐mediated complexation (Pathway 2). In addition, the high‐resolution S 2p XPS spectra exhibit a discernible binding energy shift before and after piezoelectric uranium extraction. For the pristine 3R‐ZnIn_2_S_4_ nanosheets, the S 2p_3/2_ and S 2p_1/2_ peaks were located at 161.6 and 162.7 eV, respectively. After uranium immobilization, these peaks shifted positively to 161.8 and 163.0 eV (Figure 5b). Meanwhile, a new peak emerges at approximately 170.0 eV, which is likely associated with the formation S─O species [47]. The overall positive binding energy shifts in S, In, and Zn (Figure S19 and Figure 5b) suggest a decrease in electron density around these sites, which can be attributed to uranium adsorption and the associated charge redistribution during the piezoelectric extraction process. Furthermore, XRD analysis (Figure 5c) confirms the presence of UO_2_(O_2_)·2H_2_O (PDF#16‐0207), supporting the occurrence of sequential piezoelectric reduction of H_2_O_2_‐mediateed oxidative precipitation during uranium extraction. TEM images of 3R‐ZnIn_2_S_4_ after piezoelectric extraction treatment (Figure 5d) exhibit well‐preserved nanosheet morphology. The corresponding HRTEM images display two distinct lattice fringes with spacings of 0.186 nm (Figure 5e) and 0.193 nm (Figure 5f), which can be indexed to the (114) plane of UO_2_(O_2_)·2H_2_O (PDF#16‐0207) and the (110) plane of 3R‐ZnIn_2_S_4_ (PDF#72‐0304), respectively. EDX elemental mapping demonstrates homogeneous distribution of S, Zn, In, and U throughout the nanosheets, in accordance with the morphology observed in the HAADF‐STEM image (Figure 5g). Collectively, these comprehensive characterizations not only validate the efficient uranium extraction by 3R‐ZnIn_2_S_4_ under piezocatalytic conditions, but also furnish direct and substantial evidence for the proposed dual‐pathway mechanism, elucidating the phase evolution of uranium species and the interfacial charge redistribution process during piezocatalytic uranium immobilization.

## 3 Conclusion

In summary, we demonstrate that the ABCABC stacking sequence along the *c*‐axis in 3R‐ZnIn_2_S_4_ nanosheets induces intrinsic non‐centrosymmetry. This unique structural feature enables efficient piezocatalytic uranium extraction via a dual‐coordination mechanism, which integrates direct piezoelectric reduction with in situ H_2_O_2_‐mediated complexation. Beyond the superior performance metrics exhibited by the 3R‐ZnIn_2_S_4_ nanosheets, including high extraction capacity (763.2 mg g^−1^), excellent stability, and strong antifouling efficacy, our findings provide three broader insights. First, stacking engineering in layered chalcogenides represents a promising strategy to break inversion symmetry and unlock intrinsic piezoelectricity, thereby expanding the design paradigm of piezocatalytic materials. Second, piezocatalysis directly harvests mechanical energy, overcoming the light dependence of photocatalysis and enabling effective operation under low‐light or light‐free conditions (e.g., cloudy days and deep‐sea regions). Third, the established structure–property–application framework, correlating stacking sequence, crystal symmetry, piezoelectric response, and piezocatalytic activity, provides a theoretical guideline for designing high‐performance piezocatalysts. Overall, this study advances the fundamental understanding of stacking‐induced piezocatalysis and offers a valuable reference for developing high‐efficiency piezocatalytic materials for sustainable uranium extraction and mechanical energy harvesting from aqueous environments.

## 4 Materials and Characterizations

All chemical reagents were purchased from commercial suppliers and used without further purification. The 3R‐ZnIn_2_S_4_ nanosheets were synthesized via ultrasonic liquid‐phase exfoliation of ZnIn_2_S_4_ single crystals grown by CVT with iodine as the transport agent. Detailed experimental protocols and characterization methodologies are available in the Supporting Information.

## Author Contributions

**Song Li**: methodology, investigation, software, validation, formal analysis, data curation, visualization, writing – original draft. **Huaijuan Zhou**: conceptualization, methodology, funding acquisition, writing – review and editing, supervision, project administration. **Yingting Yang**: investigation. **Yanhong Lv**: supervision. **Zdeněk Sofer**: resources, funding acquisition. **Jianyun Zheng**: investigation. **Jinhua Li**: conceptualizaiton, methodolgy, funding acquisition, writing – review and editing, supervision.

## Conflicts of Interest

The authors declare no conflicts of interest.

## Supporting information




**Supporting File**: advs76617‐sup‐0001‐SuppMat.docx.

## Data Availability

Data is available from the corresponding authors upon reasonable request.
