# Supplementary material for: ABCABC Stacking‐Enabled Non‐Centrosymmetry 3R‐ZnIn2S4 Nanosheets for Piezocatalytic Uranium Extraction
Source: Adv Sci (Weinh). 2026 Jul 16:e76617. Online ahead of print. doi: 10.1002/advs.76617 (PMC13373896; doi:10.1002/advs.76617)
Supplement: Supplementary file 1 — Supporting File: advs76617‐sup‐0001‐SuppMat.docx. [file ADVS-9999-e76617-s001.docx]

Supporting Information

**ABCABC Stacking-Enabled Non‑Centrosymmetry 3R-ZnIn_2_S_4_ Nanosheets for Piezocatalytic Uranium Extraction**

Song Li^1^, Huaijuan Zhou^1, 2, *^, Yingting Yang^1^, Yanhong Lv^3, *^, Zdeněk Sofer^4^, Jianyun Zheng^5^, Jinhua Li^2, *^

^1^ School of Materials Science and Engineering, School of Interdisciplinary Science, Beijing Institute of Technology, Beijing 100081, China.

^2^ Beijing Key Laboratory of Intelligent Molecular Materials and High-throughput Manufacturing, School of Chemistry and Chemical Engineering, Beijing Institute of Technology, Beijing, 100081, China.

^3^ School of Physics and Chemistry, Hunan First Normal University, Changsha 410205, China.

^4^ Department of Inorganic Chemistry, University of Chemistry and Technology Prague, Technicka 5, 166 28 Prague 6, Czech Republic.

^5^ State Key Laboratory of Chem/Bio-Sensing and Chemometrics, College of Chemistry and Chemical Engineering, Hunan University, Changsha 410082, China.

Correspondence: Huaijuan Zhou ([huaijuan.zhou@bit.edu.cn](mailto:huaijuan.zhou@bit.edu.cn)), Yanhong Lv ([lyh@hnfnu.edu.cn](mailto:lyh@hnfnu.edu.cn)) and Jinhua Li ([lijinhua@bit.edu.cn](mailto:lijinhua@bit.edu.cn))

1. **Materials and Characterizations**
   1. **Materials**

All chemicals were used as received without further purification. Zinc (Zn) powder (99.999%, 100 mesh) and sulfur (S) powder (99.999%, 100 mesh) were purchased from Alfa Aesar. Indium (In) powder (99.99%, 100 mesh), potassium iodide (KI), potassium hydroxyl phthalate (C_8_H_5_KO_4_), and uranyl nitrate (UO_2_(NO_3_)_2_·6H_2_O) were obtained from Aldrich. Iodine (I_2_, 99.999%) was purchased from Fisher Scientific.

- 1. **Preparation of 3R phase ZnIn_2_S_4_**

Bulk 3R-ZnIn_2_S_4_ single crystals were synthesized via the chemical vapor transport (CVT) method. Stoichiometric amounts of high-purity Zn, In, and S powders, corresponding to a targeted yield of 16 g of ZnIn_2_S_4_, were homogeneously mixed with 0.8 g of I_2_ (as the transport agent). The mixture was sealed in a quartz ampoule (40 × 250 mm) under high vacuum conditions (< 1 × 10^-3^ Pa)using an oxyhydrogen torch, with the vacuum environment achieved by an oil diffusion pump integrated with a liquid‑nitrogen cold trap to ensure ultra-high vacuum quality. Prior to the crystal growth process, the sealed ampoule was heated in a muffle furnace under a precisely programmed temperature protocol: It was first ramped to 500 °C at a heating rate of 1 °C/min and held at this temperature for 50 h, followed by further incubation at 600 °C for an additional 50 h to ensure the complete precursor reaction. For the CVT growth step, the ampoule was transferred to a two-zone tube furnace. Initially, the source zone and growth zone were maintained at 800 °C and 700 °C, respectively, for 2 days. Subsequently, the temperature gradient was reversed: the source zone temperature was gradually raised to 780 °C while the growth zone temperature was kept at 720 °C, and this temperature configuration was maintained for 10 days to facilitate crystal growth. After the ampoule was naturally cooled to room temperature, well‑faceted bulk crystals were collected from the growth zone and repeatedly rinsed with acetone to eliminate residual surface iodine, yielding phase‑pure bulk 3R-ZnIn_2_S_4_. For the preparation of few-layered 3R- ZnIn_2_S_4_ nanosheets, the as‑synthesized bulk crystals were dispersed in a 1:1 (v/v) mixed solvent of ethanol and ultrapure water (20 mL each) and subjected to ultrasonic exfoliation for 12 h under ambient conditions. The exfoliated product was then collected by centrifugation, thoroughly washed with ethanol and ultrapure water, and dried in a vacuum oven at 50 °C for 6 h to afford few-layer 3R-ZnIn_2_S_4_ nanosheets.

- 1. **Characterizations**

The microstructural and morphological features of as-synthesized samples were characterized using a Talos F200X transmission electron microscope (Thermo Fisher Scientific, USA), encompassing transmission electron microscopy (TEM), high-resolution transmission electron microscopy (HRTEM), high-angle annular dark-field scanning transmission electron microscopy (HAADF-STEM), selected area electron diffraction (SAED), and energy-dispersive X-ray spectroscopy (EDX) elemental mapping for elemental distribution analysis. The crystal structures of 3R-ZnIn_2_S_4_ were investigated via X-ray diffraction (XRD) measurements using a Rigaku D/MAX-2550 diffractometer (Japan). Raman spectroscopy measurements were performed on a WITec alpha300R spectrometer (Germany) with an excitation wavelength of 532 nm and a laser power of 0.5 mW, which was carefully controlled to prevent thermal damage to the samples. Piezoelectric properties were characterized by piezoelectric force microscopy (PFM) using an Asylum Research Cypher S atomic force microscope (Oxford Instruments, USA), where spatially resolved amplitude and phase images of the surface piezoelectric response were derived through the processing of detected electrical signals. The UV–visible diffuse reflectance spectra (DRS) were acquired on a Shimadzu UV‑2600i spectrophotometer (Japan) using BaSO_4_ as a reference, and the spectra were utilized for optical bandgap estimation. Electron paramagnetic resonance (EPR) spectroscopy was conducted on a JEOL JES-FA200 spectrometer (Japan) to identify defects and detect free radical species in the samples. X-ray photoelectron spectroscopy (XPS) and valence band XPS (VB-XPS) measurements were carried out on a Kratos Axis Supra^+^ spectrometer (UK) with monochromated Al Kα radiation (hν = 1486.8 eV). All XPS binding energies were calibrated with respect to the adventitious carbon C 1s peak at 284.8 eV to ensure the accuracy of the chemical state analysis.

- 1. **Photoelectrochemical Measurements**

Photoelectrochemical (PEC) measurements were conducted using a CHI760e electrochemical workstation (Shanghai Chenhua Instruments Co., China) with a standard three-electrode cell configuration, wherein a 1 M aqueous K_2_SO_4_ solution was employed as the electrolyte. The working electrode was fabricated via a drop-casting method: 5 mg of the catalyst powder was dispersed in a mixed solution consisting of 950 μL isopropyl alcohol and 50 μL Nafion to form a homogeneous catalyst ink, which was then drop-cast onto a 10 × 10 mm fluorine-doped tin oxide (FTO) glass substrate and subsequently dried under an infrared lamp to ensure solvent evaporation and firm adhesion of the catalyst layer. A saturated Ag/AgCl electrode and a platinum wire were employed as the reference and counter electrodes, respectively. Transient photocurrent and piezoelectric current responses were recorded at a bias potential of 0.5 V (vs. Ag/AgCl) under chopped illumination from a 300 W Xe lamp or ultrasonic irradiation (35 kHz, 50 W), with 30 s light on/off cycles. Electrochemical impedance spectroscopy (EIS) was performed under illumination, with an AC signal amplitude of 5 mV and a frequency range spanning from 100 kHz to 0.1 Hz. Mott–Schottky analysis was carried out in a dark environment at three frequencies (1500, 2000, and 2500 Hz).

The optical band gap values of the as-synthesized samples were calculated based on the DRS data. Initially, the diffuse reflectance spectra were converted to the absorption coefficient $F(R_{\infty})$ using the Kubelka–Munk function^[1]^:

| $F(R_{\infty})=\frac{(1-R_{\infty})^{2}}{2R_{\infty}}$ | (S1) |
| --- | --- |

Where $R_{\infty}$ denotes the absolute reflectance of the sample. The obtained $F(R_{\infty})$ values were subsequently substituted into the Tauc equation to determine the optical band gap:

| $[F(R_{\infty})h\nu]^{1/n}=A(h\nu-E_{g})$ | (S2) |
| --- | --- |

Where $h\nu$ denotes the photon energy, $A$ is a proportionality constant, and $E_{g}$ is the optical band gap of the sample. The exponent n depends on the nature of the optical transition, with n = 2 for indirect allowed transitions and n = 1/2 for direct allowed transitions. The optical bandgap of 3R-ZnIn_2_S_4_ nanosheets was calculated to be 2.41 eV by plotting $[F(R_{\infty})h\nu]^{1/n}$ versus $h\nu$ and extrapolating the linear segment of the resulting curve to the x-axis (where $[F(R_{\infty})h\nu]^{1/n}$=0).

The carrier density (*N*_d_) and flat-band potential (*E*_FB_) of the samples were determined through Mott-Schottky analysis. For an n-type semiconductor, the relationship between the space-charge layer capacitance (C) and the applied potential (E) is described by the following equation (S3)^[2]^:

| $\frac{1}{C^{2}}$ = $\frac{2}{N_{d}\varepsilon_{0}\varepsilon_{r}e}$(E - E*_FB_* -$\frac{kT}{e}$) | (S3) |
| --- | --- |

where C is the space-charge capacitance per unit area (F/m^2^), e is the electron charge (1.602 × 10^-19^ C), ε_r_ is the relative permittivity, ε_0_ is the permittivity of the vacuum (8.854 × 10^-12^ F/m), *N*_d_ is carrier density (m^-3^), *E* is the applied potential, k is the Boltzmann constant, and T is the absolute temperature. The flat-band potential ($E_{fb}$) was estimated by extrapolating the linear portion of the $1/C^{2}$ vs. $E$ plot to the intercept where $1/C^{2}=0$.

Rotating disk electrode (RDE) measurements were performed on a Pine AFMSRXE 1523 electrochemical system using a three-electrode configuration, with a saturated Ag/AgCl reference electrode and a platinum wire as the counter electrode. The working electrode was prepared as follows: 5 mg of the 3R ZnIn_2_S_4_ powder was dispersed in 1 mL of anhydrous ethanol containing 25 μL of Nafion solution to form a homogeneous catalyst ink. A 10 μL aliquot of this ink was then drop-cast onto the surface of RDE (geometric area: 0.20 cm^2^) and dried naturally at room temperature to form a uniform catalyst film. Linear sweep voltammetry (LSV) tests were conducted in O_2_‑saturated phosphate-buffered saline (PBS, 1 M, pH = 6.8 ± 0.2) at a scan rate of 10 mV/s. The average electron transfer number (n) for the oxygen reduction reaction (ORR) was calculated from the slopes of Koutecký–Levich (K–L) plots using the following equations^[3,4]^:

| $\frac{1}{j}=\frac{1}{j_{k}}+\frac{1}{B\omega^{1/2}}$ | (S4) |
| --- | --- |
| $B=0.62nFCD^{2/3}\nu^{-1/6}$ | (S5) |

where $j$ is the measured current density (mA/cm^2^), $j_{k}$ is the kinetic current density (mA/cm^2^), $\omega$ is the rotation speed (rpm), $F$ is the Faraday constant (96485 C·mol^-1^), $C$ is the bulk concentration of O_2_ in water (1.26 × 10^-3^ mol·cm^-3^), $D$ is the diffusion coefficient of O_2_ (2.7 × 10^-5^ cm^2^·s^-1^), and $\nu$ is the kinetic viscosity of water (0.01 cm^2^·s^-1^) at 298K.

- 1. **Piezocatalytic H_2_O_2_ Production**

Piezocatalytic H_2_O_2_ production experiments were carried out in a 100 mL quartz beaker, which was vertically fixed at the central position of an ultrasonic cleaner (35 kHz, 50 W) to ensure uniform ultrasonic irradiation through the reaction system. Prior to the reaction, all glassware was thoroughly cleaned with deionized water, soaked in 10% nitric acid for 24 h to remove metal ion contaminants, and subsequently rinsed repeatedly with ultrapure water before drying in an oven at 60 °C for 2 h to ensure no residual impurities.

In a typical experimental procedure, 20 mg of the pretreated catalyst was accurately weighed and dispersed into 50 mL of ultrapure water in a quartz beaker. Before initiating the reaction, the catalyst suspension was stirred for 30 min to achieve homogeneous dispersion and establish adsorption-desorption equilibrium between the catalyst and water. The ultrasonic cleaner was then activated to trigger the piezocatlytic reaction, with ultrasonic vibration serving as the sole stirring source to maintain the catalyst in uniform suspension. A circulating water bath was connected to the ultrasonic cleaner to maintain the reaction temperature at a constant 25±0.5°C, thereby eliminating temperature fluctuations as a confounding variable. The total reaction duration was set to 150 min, and 2 mL liquid aliquots were withdrawn at 30 min intervals (i.e., 0, 30, 60, 90, 120, and 150 min). The 0 min aliquot was collected immediately after catalyst dispersion and prior to ultrasonic irradiation, serving as the blank control to account for any background H_2_O_2_. Each withdrawn aliquot was promptly filtered through a 0.22 μm hydrophilic polyethersulfone (PES) membrane syringe filter to remove catalyst particles; the initial 0.5 mL of the filtrate was discarded to minimize interference from catalyst adsorption on the membrane, and the subsequent filtrate was collected into a pre-cleaned sampling vial for H_2_O_2_ concentration quantification.

The concentration of H_2_O_2_ in the filtrate was quantified via the iodometric titration method coupled with UV-visible spectrophotometry. Prior to measurement, a TU-1901 UV-visible spectrophotometer was preheated for 30 min and calibrated with blank ultrapure water at a detection wavelength of 350 nm to ensure measurement accuracy. The iodometric detection system was prepared by mixing 1 mL of filtrate with 1 mL of 0.4 M KI solution and 1 mL of 0.1 M C_8_H_5_KO_4_ buffer solution. The mixture was incubated in the dark for 30 min to ensure complete redox reaction between H_2_O_2_ and I^-^ (reaction equation: H_2_O_2_ + 2I^-^ + 2H^+^ → I_2_ + 2H_2_O). The absorbance of the generated I_2_ was measured at 351 nm, with three parallel measurements performed for each sample to reduce random errors. The H_2_O_2_ concentration was calculated based on a pre-established calibration curve of I_2_ absorbance versus H_2_O_2_ concentration.

To identify the dominant reactive species and elucidate the H_2_O_2_ formation pathway, systematic radical quenching experiments were conducted. Typically, a series of sacrificial agents was separately introduced into the piezocatalytic reaction system under identical conditions. Specifically, p-benzoquinone (p-BQ, 1 mM) was used as a superoxide radical (•O_2_^⁻^) scavenger, triethanolamine (TEOA, 10 mM) as a hole (h^+^) scavenger, tert-butanol (TBA, 10 mM) as a hydroxyl radical (•OH) scavenger, and sodium bromate (NaBrO_3_, 10 mM) as an electron (e^⁻^) trapping agent. Each scavenger was added to the reaction solution prior to ultrasonic agitation (35 kHz, 50 W). The H_2_O_2_ concentration was measured at regular intervals following the same protocol as described above. The inhibition efficiency of each scavenger was evaluated by comparing the H_2_O_2_ yield with that of the control experiment (without any scavenger). All experiments were performed in triplicate to ensure reproducibility.

- 1. **Piezocatalytic Uranium Extraction**

Piezocatalytic uranium extraction experiments were conducted by reacting 50 mL of U(VI) aqueous solutions (initial concentration: 10–200 ppm) with 5 mg of the as-synthesized catalyst. All reactions were performed under ultrasonic irradiation (35 kHz, 50 W) using an ultrasonic cleaner, with samples collected at 30 min intervals for subsequent U(VI) concentration analysis. After one extraction cycle, the catalyst was recovered by centrifugation, thoroughly washed with ultrapure water to remove adsorbed species, and reused for up to 5 consecutive cycles to evaluate its reusability and stability.

To evaluate the anti-interference capability of the catalyst under realistic wastewater conditions, uranium extraction experiments were conducted in aqueous solutions containing U(VI) and various competing metal ions (V^5+^, Zr^4+^, Fe^3+^, Co^2+^, Ni^2+^, Cu^2+^, Zn^2+^, and K^+^) simultaneously. The initial concentration of U(VI) was fixed at 50 ppm, and each competing ion was also maintained at 50 ppm to simulate the composition of actual uranium-containing wastewater conditions. Quantitative elemental analysis of U(VI) and competing metal ions was performed using an inductively coupled plasma optical emission spectrometer (ICP-OES, PerkinElmer 2100, USA).

The uranium reduction efficiency (n, %) and the equilibrium extraction capacity ($q_{e}$, mg/g) were calculated using Equations S6 and S7, respectively:

| $n=\frac{(C_{0}-C_{e})}{C_{0}}$ | (S6) |
| --- | --- |

where $C_{0}$ (mg/L) and $C_{e}$ (mg/L) are the initial and equilibrium concentrations of U(VI) in the aqueous solution, respectively.

| $q_{e}=\frac{\left( C_{0}-C_{e} \right) V}{m}$ | (S7) |
| --- | --- |

where $C_{0}$ (mg/L) and $C_{e}$ (mg/L) are the initial and equilibrium concentrations of U(VI), respectively; $V$ (L) is the volume of the reaction solution; and $m$ (g) is the mass of the catalyst.

Moreover, the extraction kinetics of uranium were fitted using the Pseudo-first-order and the pseudo-second-order kinetic models, described by Equations S8 and S9:

| $Q_{t}=Q_{e}(1-e^{-K_{1}t})$ | (S8) |
| --- | --- |

where $Q_{e}$(mg/g) is the equilibrium extraction capacity, $Q_{t}$ (mg/g) is the extraction capacity at time t (min), and $K_{1}$ is a pseudo-first-order adsorption kinetics constant.

| $\frac{t}{Q_{t}}=\frac{t}{Q_{e}}+\frac{1}{K_{2}Q_{e}^{2}}$ | (S9) |
| --- | --- |

where $Q_{e}$(mg/g) is the equilibrium extraction capacity, $Q_{t}$ (mg/g) is the extraction capacity at time t, and $K_{2}$ is a pseudo-second-order adsorption kinetics constant. The fitting goodness of the kinetic models was evaluated using the correlation coefficient (R^2^).

**1.7 Anti-biofouling Studies**

The anti-biofouling performance of 3R-ZnIn_2_S_4_ against marine-relevant microorganisms was evaluated using *Staphylococcus aureus* (*S. aureus*) and *Escherichia coli* (*E. coli*) as model strains, aiming to simulate its resistance to biofilm formation in complex seawater environments. The assessment was performed in accordance with a standard catalytic antibacterial protocol to ensure experimental reproducibility and comparability. All materials and equipment involved in the experiments were first sterilized under ultraviolet (UV) irradiation for 30 min in a biosafety cabinet to eliminate background microbial contamination. Subsequently, 20 mg of the as-synthesized 3R-ZnIn_2_S_4_ catalyst was accurately weighed and dispersed into 25 mL of bacterial suspension (initial concentration: ~10^8^ colony-forming units per milliliter, CFU/mL).

The bacterial-catalyst suspension was then exposed to ultrasonic irradiation (35 kHz, 50 W) using an ultrasonic cleaner for a total duration of 2 hours, with a circulating water bath employed to maintain the reaction temperature at 25 ± 0.5 °C to avoid thermal effects on bacterial viability. After the piezocatalytic treatment, 100 μL of the suspension was collected and serially diluted 10,000-fold with sterile water to ensure the number of surviving bacterial colonies fell within the countable range. Aliquots (100 μL) of the diluted suspension were spread evenly on Luria-Bertani (LB) agar plates following the guidelines outlined in the National Standard of China GB/T 4789.2^[5]^. The inoculated plates were incubated at 37 °C for 18 hours under aerobic conditions, after which the number of viable bacterial colonies was counted. A control group (without catalyst addition, subjected to the same ultrasonic irradiation and incubation conditions) was set up in parallel to exclude the antibacterial effect of ultrasonic irradiation alone. The antibacterial efficiency (R) was quantitatively evaluated using Equation S10:

| R (%) = (A – B) / A × 100%, | S10 |
| --- | --- |

where A and B represent the viable bacterial counts (CFU/mL) of the control group and the piezocatalytic treatment group, respectively. All experiments were performed in triplicate, and the results were expressed as mean ± standard deviation (SD) to ensure statistical reliability.

1. **Density Functional Theory (DFT) Calculations**

All of the calculations were performed within the framework of the spin-polarized density functional theory, employing the projector augmented plane-wave (PAW) method as implemented in the Vienna ab initio simulation package (VASP)^[6,7]^. The generalized gradient approximation (GGA) with the Perdew-Burke-Ernzerhof (PBE) exchange-correlation functional was adopted to describe the electron exchange-correlation potential^[8,9]^. Long-range van der Waals (vdW) interactions were accounted for using the DFT-D3 dispersion correction approach^[10]^. The plane-wave cut-off energy was set to 480 eV to ensure sufficient convergence of the electronic wavefunctions. In the iterative solution of the Kohn-Sham equation, an energy convergence criterion of 10^−4^ eV was imposed. All atomic structures were fully relaxed until the residual forces on each atom were reduced to less than 0.05 eV/Å, guaranteeing the structural stability of the optimized configurations. Data analysis and structural visualization were conducted using the VASPKIT code^[11]^ and VESTA software^[12]^, respectively. To eliminate interlaminar interactions between adjacent slabs in the periodic supercell, a vacuum spacing of 20 Å was introduced perpendicular to the slab surface.

The Gibbs free energy difference (ΔG) for reaction intermediates was defined in Equation S11:

| $\Delta G=\Delta E+\Delta E_{ZPE}-T\Delta S$ | (S11) |
| --- | --- |

where ΔE is the total energy difference between the slab and respective terminations computed by DFT-PBE. ΔEZPE and TΔS denote differences in zero-point energy and entropy between adsorbed states of reaction intermediates and the gap phase, respectively. T is the room temperature (298.15 K).

The charge density difference ($\Delta\rho$) of the A/B was defined as Equation S12:

| $\Delta\rho=\rho_{\text{A/B}}-\rho_{A}-\rho_{B}$ 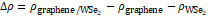 | (S12) |
| --- | --- |

where $\rho_{\text{A/B}}$, $\rho_{\text{A}}$ and $\rho_{\text{B}}$ are the charge densities of the A/B heterostructure, the isolated A slab, and the isolated B slab, respectively


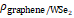

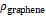

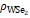


The quantity of charge transfer was quantified using Bader charge analysis.

**3. Supplementary**

**3.1 Supplementary Figures**

**
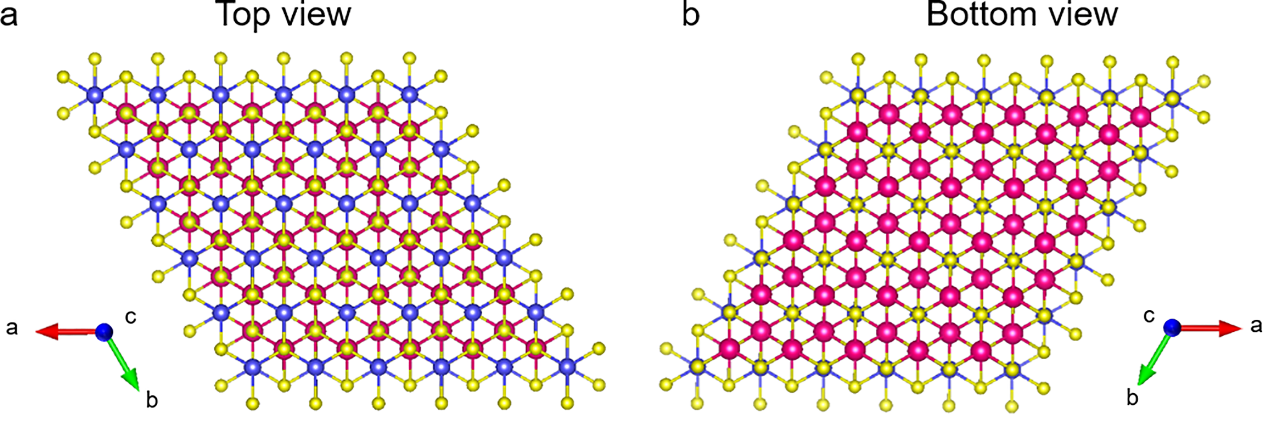
**

**Figure S1.** (a) Top view and (b) bottom view of the 3R-ZnIn_2_S_4_ crystal structure.

**
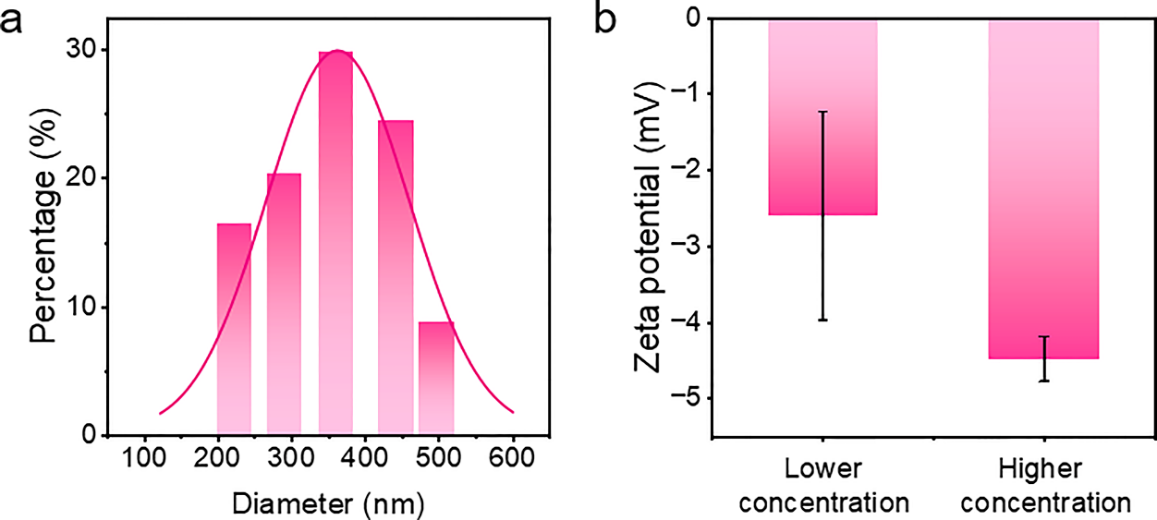
**

**Figure S2.** (a) The hydrodynamic diameters and (b) Zeta potential of various nanosheets measured by dynamic light scattering (n = 3 independent experiments).

**
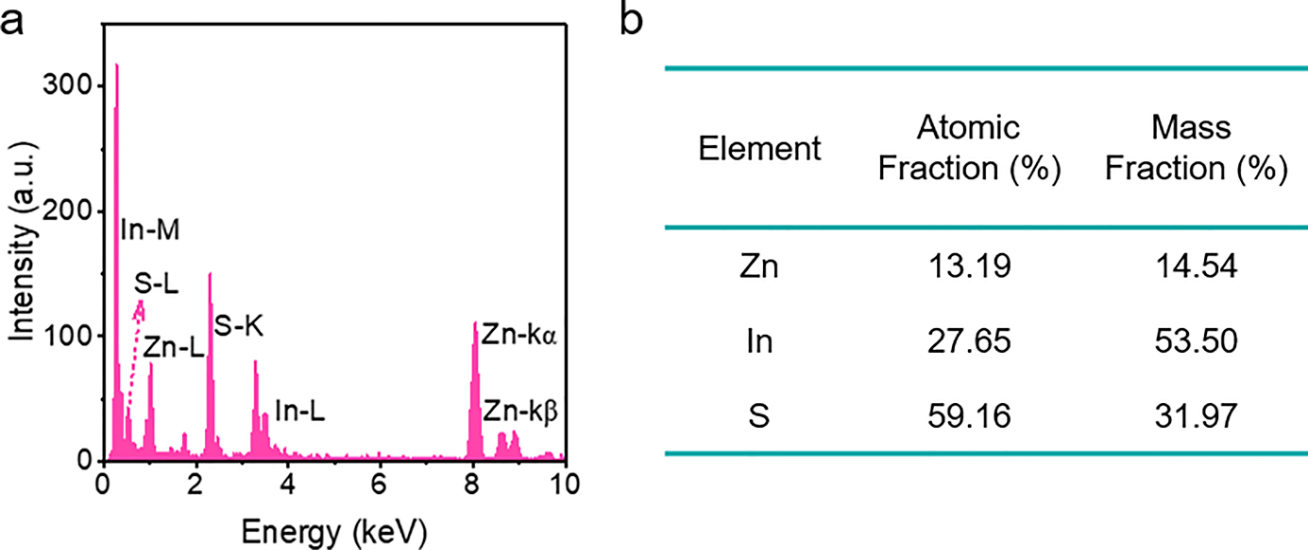
**

**Figure S3.** TEM-EDX compositional analysis.


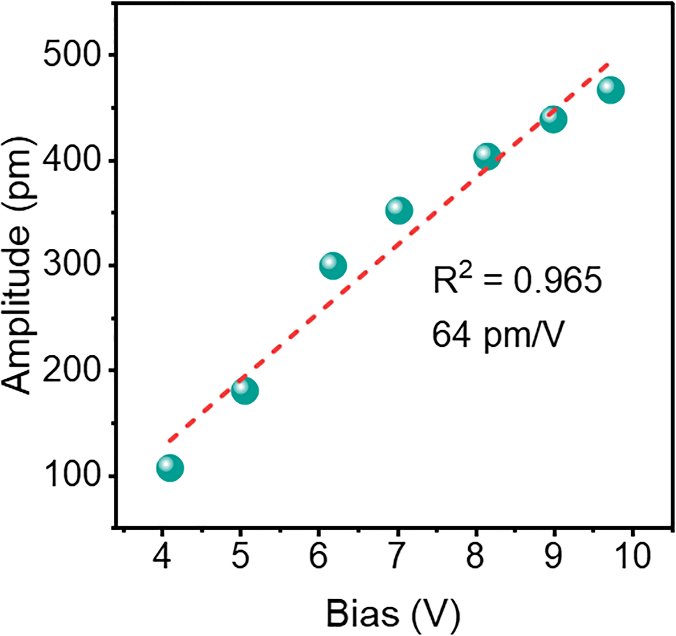


**Figure S4.** Determination of the effective piezoelectric coefficient d_33_ from the butterfly loop.

**
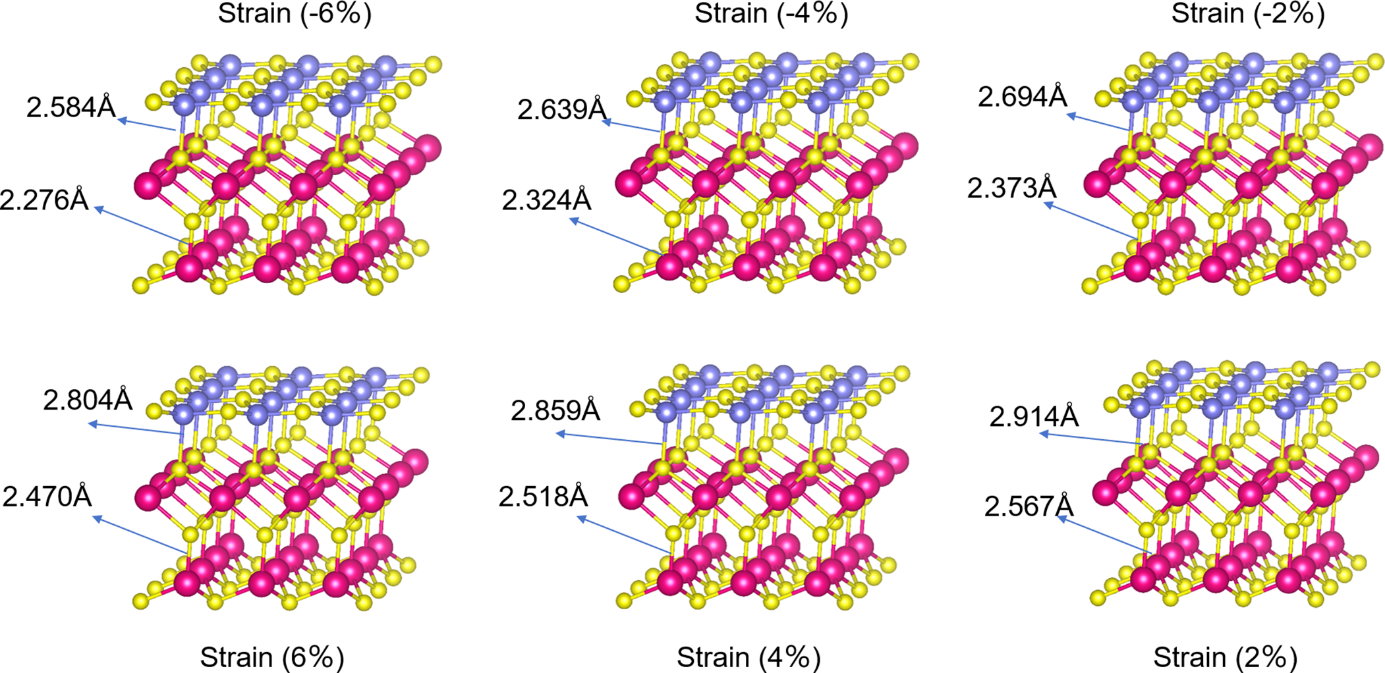
**

**Figure S5.** Calculated structural changes of 3R ZnIn_2_S_4_ under uniaxial strain along the c axis, ranging from –6% to 6% under vacuum conditions.

**
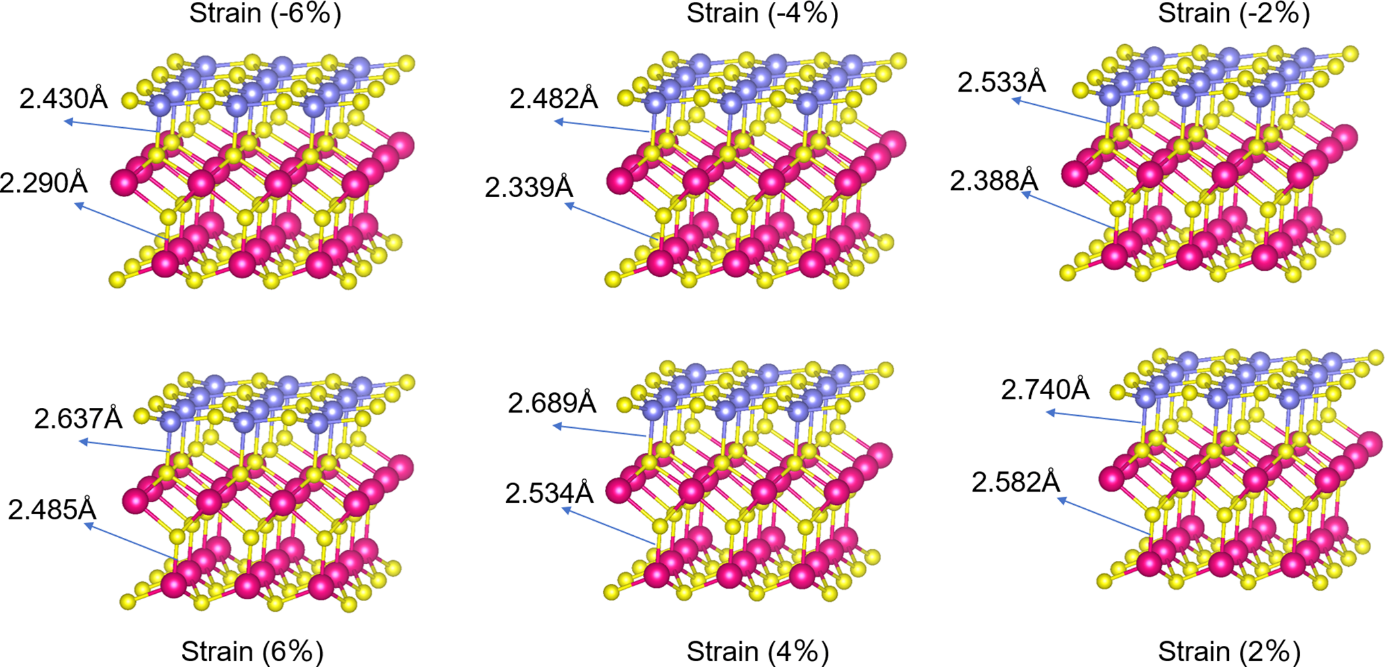
**

**Figure S6.** Calculated structural changes of 3R ZnIn_2_S_4_ under uniaxial strain along the c axis, ranging from –6% to 6%, under implicit solvation conditions.


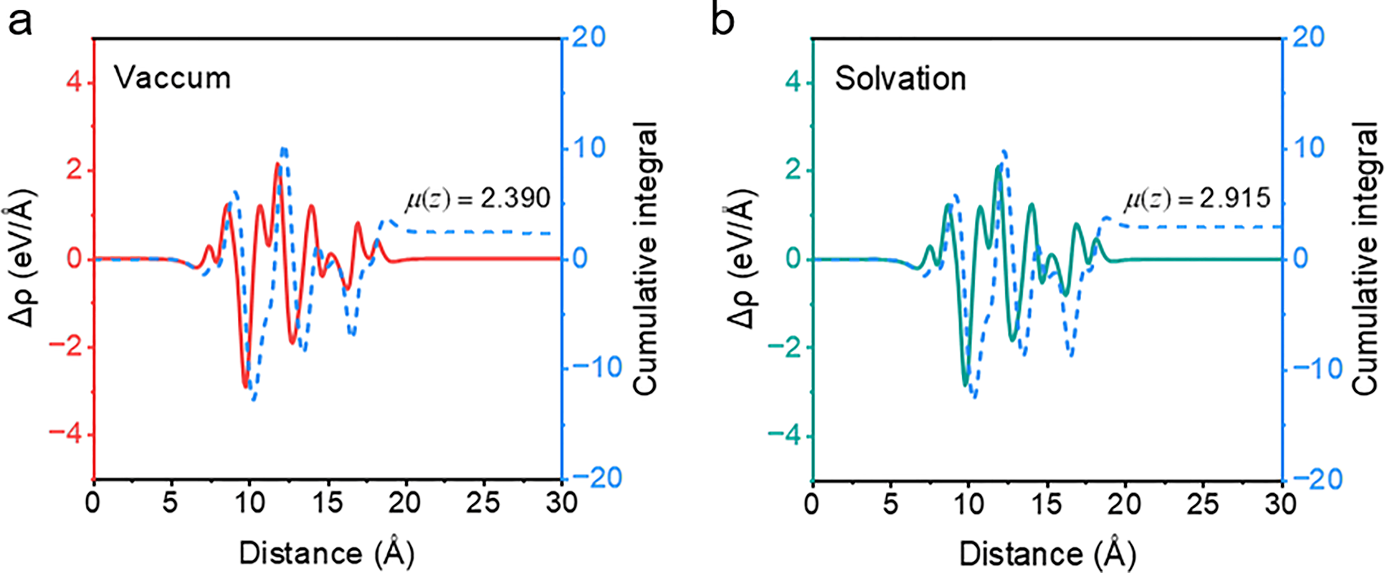


**Figure S7.** Electron density distribution along the Z-direction within the lattice of 3R ZnIn_2_S_4_ and the corresponding dipole moment calculated by integrating the electron density in (a) vacuum and (b) solvation.


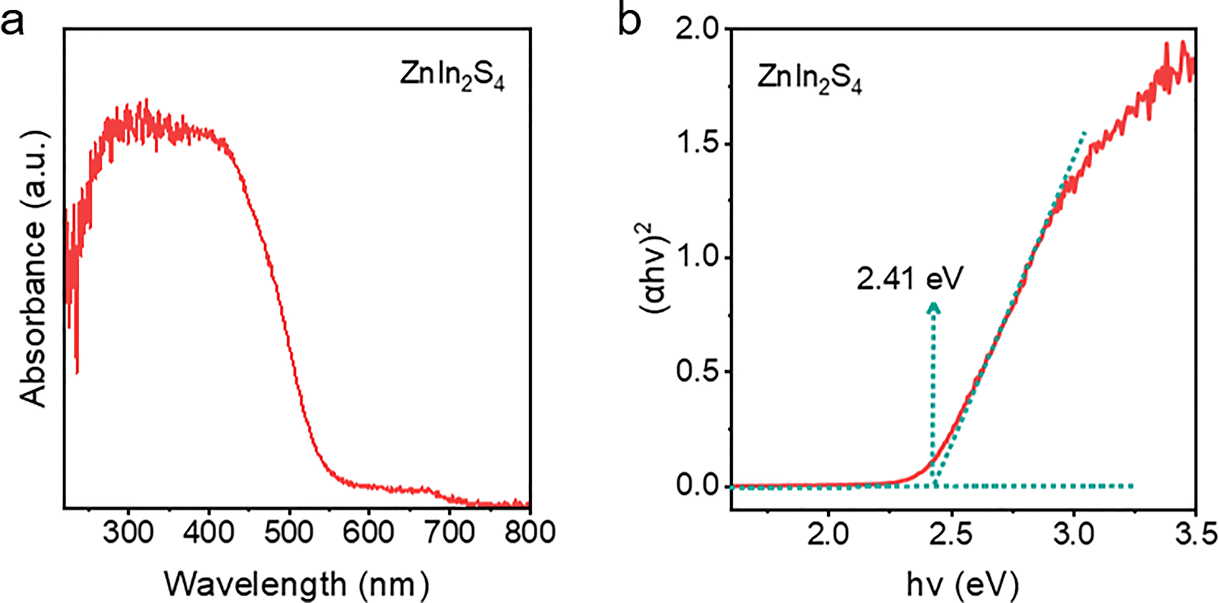


**Figure S8**. (a) UV-vis absorption spectrum of 3R ZnIn_2_S_4_ with a visible-light absorption and (b) an optical bandgap of approximately 2.41 eV (derived from Kubelka-Munk transformed reflectance and Tauc plot).


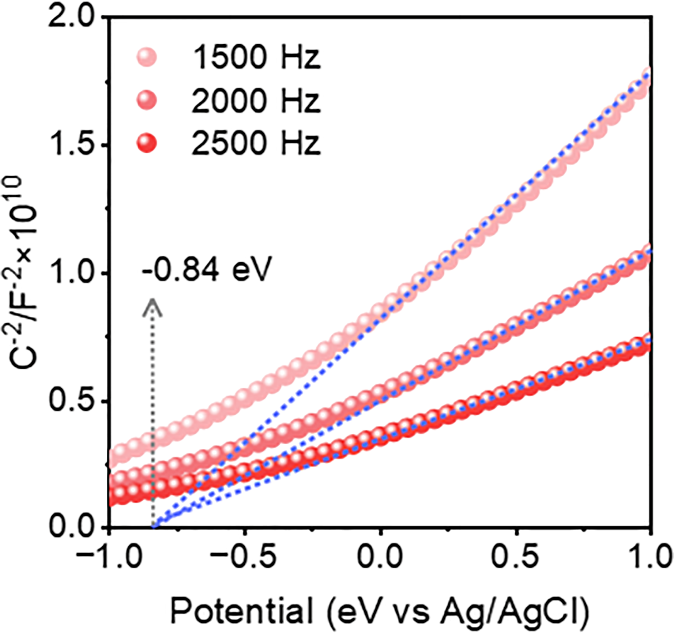


**Figure S9.** Mott–Schottky plot of 3R ZnIn_2_S_4_.


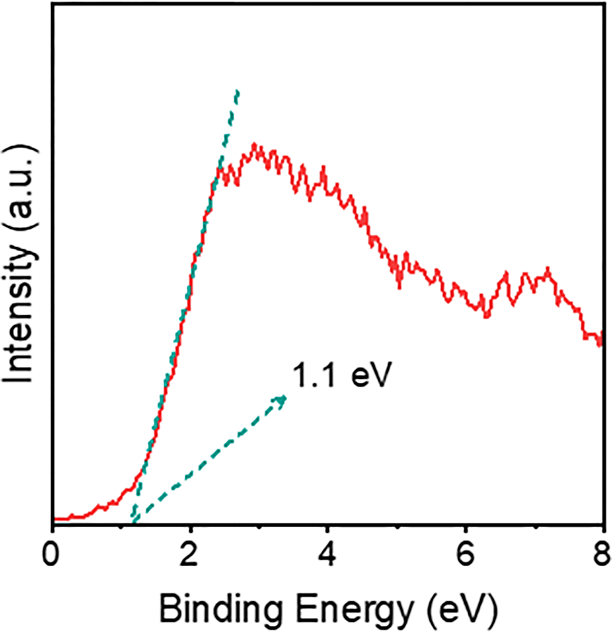


**Figure S10.** Valence band X-ray photoelectron spectroscopy (VB-XPS) of 3R ZnIn_2_S_4_.

The flat-band potential (E_fb_) was experimentally measured with respect to the saturated Ag/AgCl reference electrode and subsequently converted to the reversible hydrogen electrode (RHE) scale using the Nernst equation, as expressed below:

| E(RHE) = E(Ag/AgCl) + 0.197 + 0.059 × pH | S13 |
| --- | --- |

Based on this conversion, the E_fb_ of 3R ZnIn_2_S_4_ was calculated to be –0.23 V vs. RHE. The valence band maximum (VBM) position relative to the Fermi level (E_F_) was determined via VB-XPS, which revealed a VBM onset at 1.1 eV below E_F_. For n-type semiconductors, the flat-band potential is approximately equivalent to the Fermi level (E_fb_ ≈ E_F_) on the RHE scale^[13]^. Thus, the absolute VBM position was calculated as 0.87 eV vs. RHE (derived from 1.1 eV – 0.23 eV). Utilizing the optical bandgap (Eg = 2.41 eV) of 3R-ZnIn_2_S_4_ determined earlier, the conduction band minimum (CBM) position was further calculated using the following relationship:

| *E*_CB_ = *E*_VB_ – *E*_g_ = 0.87 eV – 2.41 eV = –1.54 eV vs. RHE. | S14 |
| --- | --- |


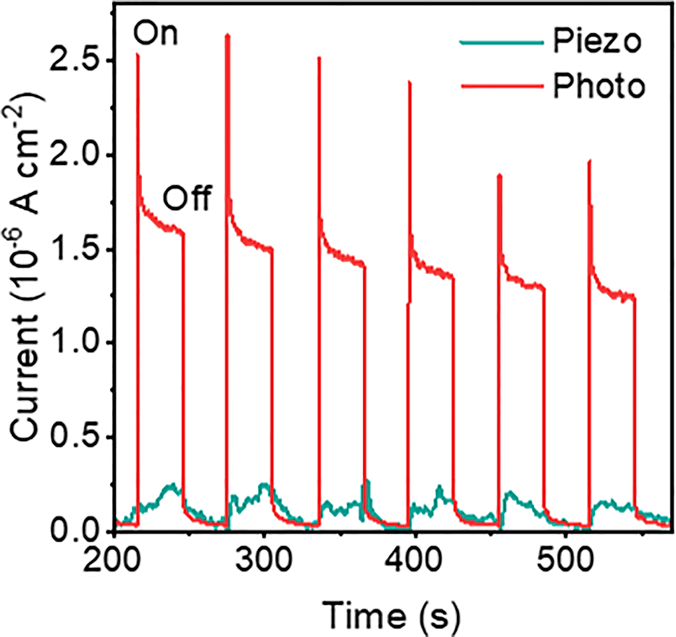


**Figure S11**. Transient photocurrent and piezoelectric current measurements of 3R ZnIn_2_S_4_.

To further corroborate the piezocatalytic potential for thermodynamically favorable H_2_O_2_ production, transient piezoelectric and photoelectric current measurements were conducted. As shown in **Figure S11**, 3R ZnIn_2_S_4_ exhibits a strong and stable photocurrent derived from efficient photon-to-electron conversion, while a distinct yet comparatively weak transient piezoelectric current is induced under mechanical strain. This piezoelectric current originates from charge displacement within the noncentrosymmetric crystal structure of 3R ZnIn_2_S_4_. Although the piezoelectric current is constrained by the low intrinsic carrier density in the dark, the detectable charge separation and transfer still confirm the thermodynamic favorability of piezocatalytic electron transfer for O_2_ reduction to H_2_O_2_, further underscoring the considerable potential of 3R ZnIn_2_S_4_ as a piezocatalyst for H_2_O_2_ evolution. These findings are further supported by field-dependent electrochemical impedance spectroscopy (EIS). As shown in **Figure S12**, the decreasing semicircle radius in the Nyquist plot with increasing bias voltage indicates a significant reduction in charge-transfer resistance, which is attributed to voltage-modulated piezoelectric polarization that facilitates charge separation and transfer at the catalyst–electrolyte interface. This reduced charge-transfer resistance lowers the kinetic and thermodynamic barriers for ORR processes, further confirming that 3R ZnIn_2_S_4_ enables thermodynamically favorable H_2_O_2_ evolution via piezocatalysis.


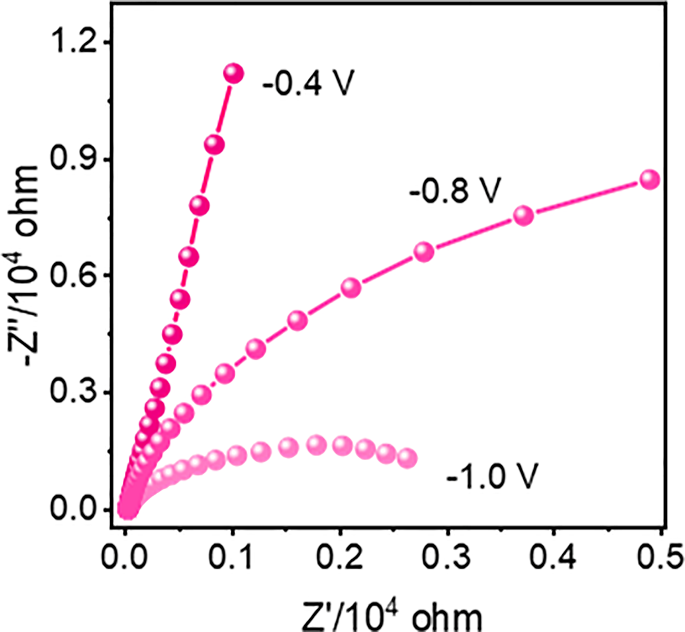


**Figure S12**. Bias-dependent Nyquist plots of ZnIn_2_S_4_.


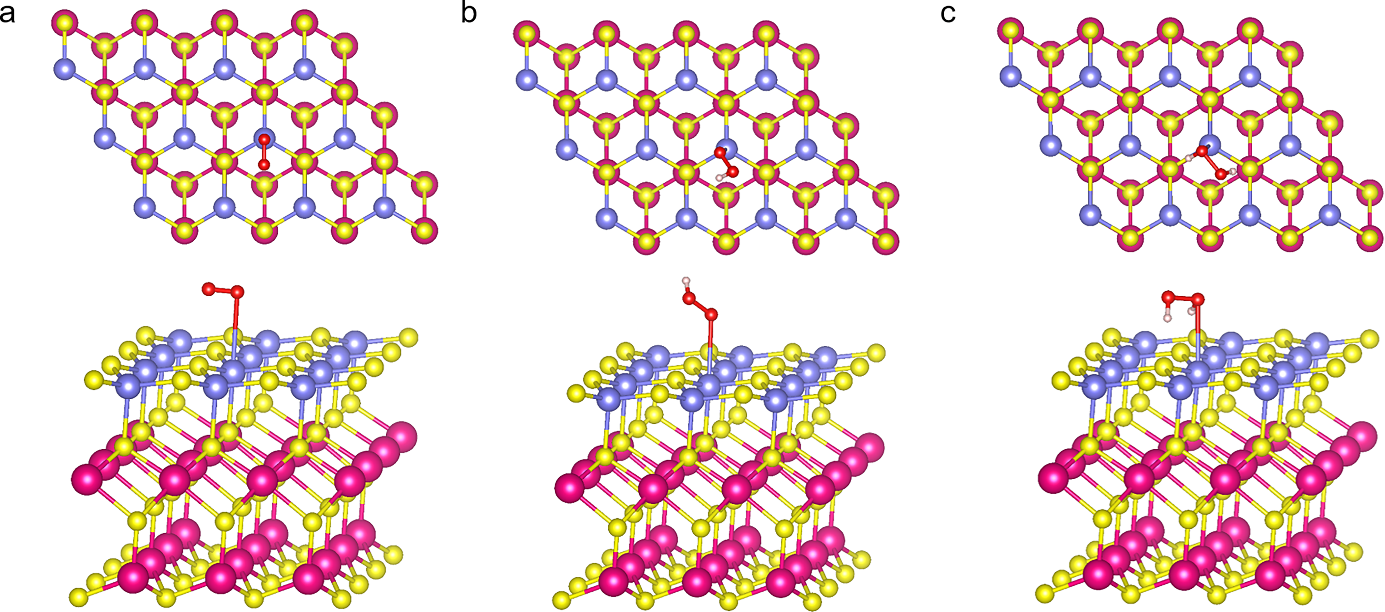


**Figure S13**. Optimized adsorption configurations of key ORR intermediates (O_2_, *OOH, *HOOH) on the (110) surface of 3R ZnIn_2_S_4_ under solvation conditions.


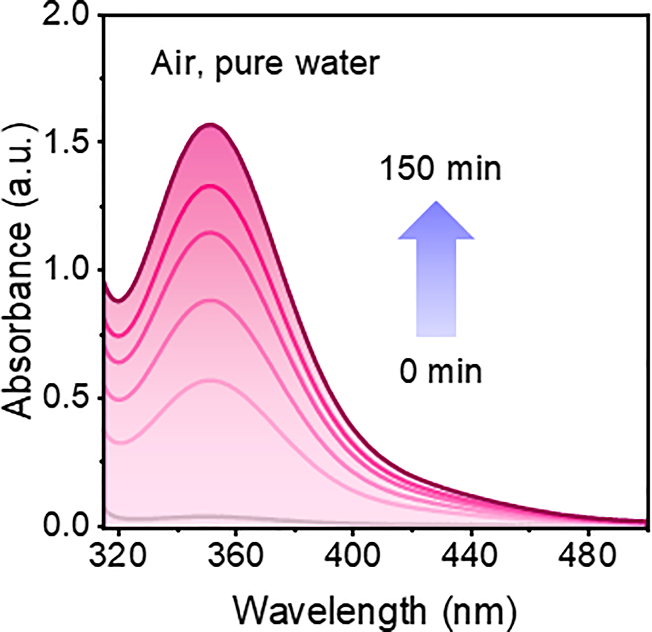


**Figure S14**. Time-dependent UV–vis absorption spectra of the H_2_O_2_ detection solution under ultrasonic irradiation (35kHz, 50W) in pure water.


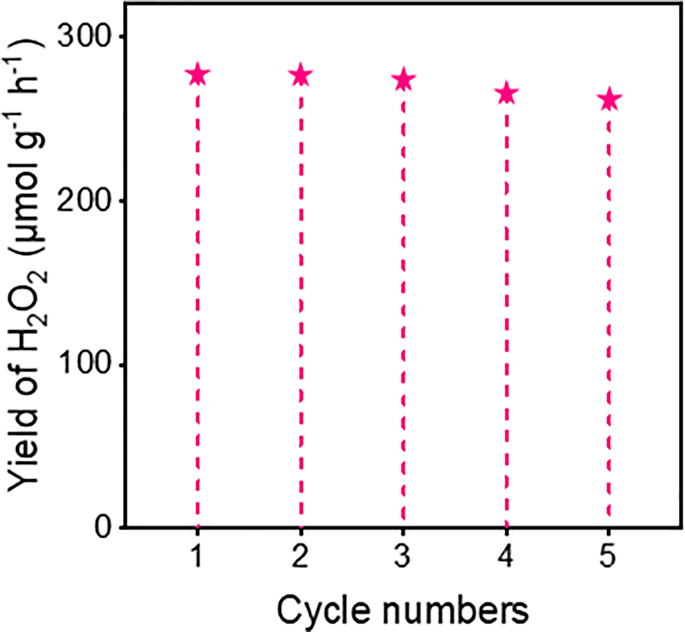


**Figure S15.** Cycling performance of 3R ZnIn_2_S_4_ for piezocatalytic H_2_O_2_ production over five consecutive cycles.


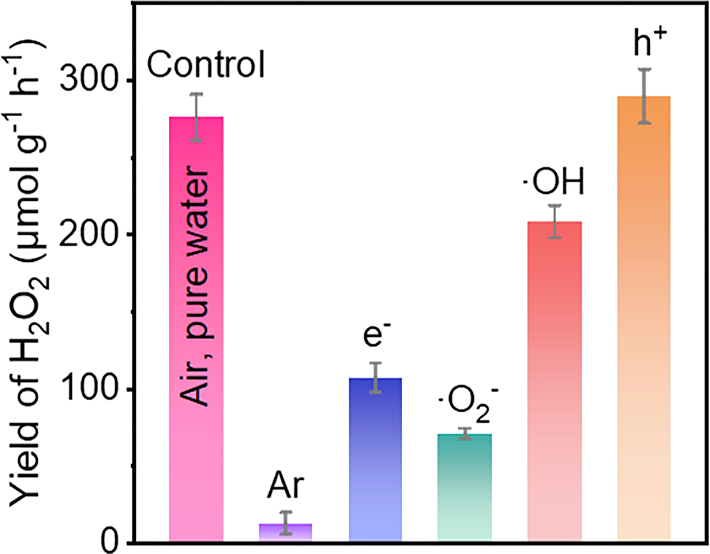


**Figure S16.** Radical quenching experiments for piezocatalytic H_2_O_2_ generation over 3R ZnIn_2_S_4_ under ultrasonication, using various sacrificial agents: Ar purging (30 min to remove dissolved oxygen), p-BQ (•O_2_^⁻^ scavenger), TEOA (hole scavenger), TBA (•OH scavenger), and NaBrO_3_ (electron scavenger).

.


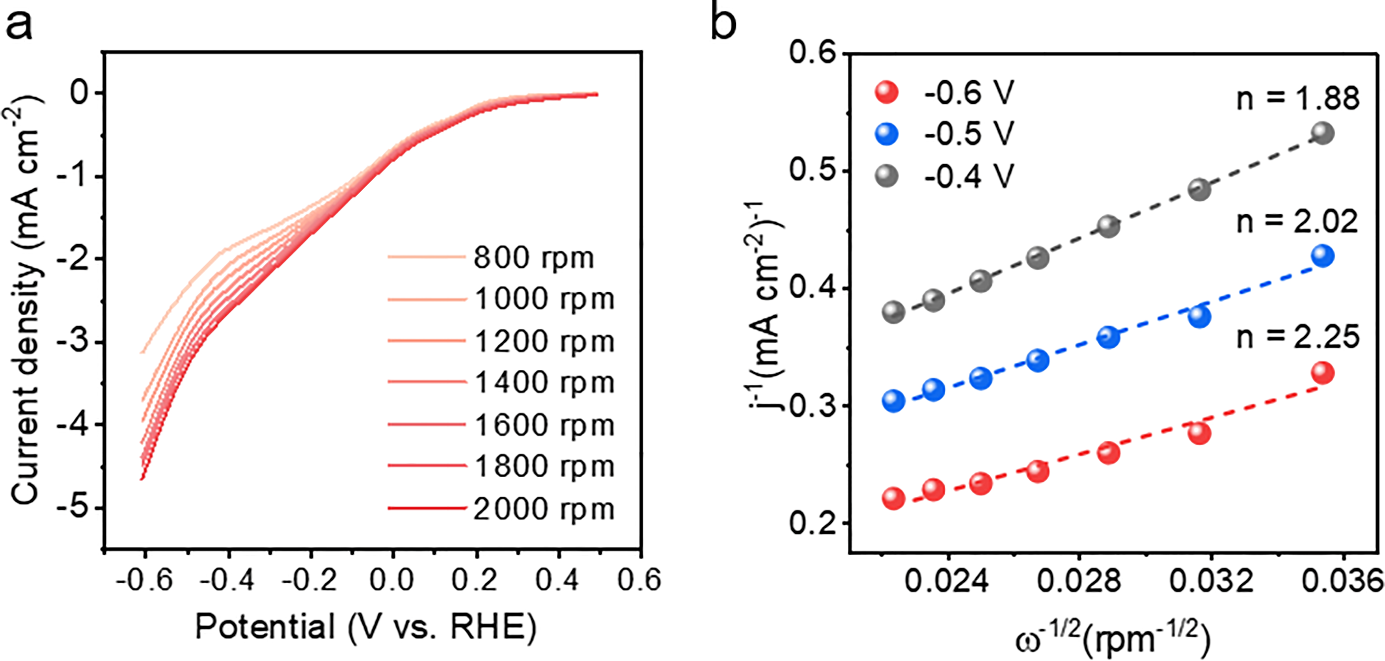


**Figure S17**. Rotating disk electrode (RDE) measurements for 3R-ZnIn_2_S_4_. (a) Linear sweep voltammetry (LSV) curves at different rotation speeds (400–2500 rpm) in O_2_-saturated PBS solution. (b) Corresponding Koutecký–Levich (K–L) plots at various potentials. The calculated average electron transfer number is approximately 2.05, confirming the dominant two-electron ORR pathway for H_2_O_2_ generation.

To quantitatively determine the electron transfer number during oxygen reduction, rotating disk electrode (RDE) measurements were performed on 3R-ZnIn_2_S_4_ in O_2_‑saturated phosphate‑buffered saline (PBS, 1 M, pH = 6.8 ± 0.2) at various rotation speeds. Linear sweep voltammetry (LSV) was conducted at a scan rate of 10 mV/s using a three‑electrode configuration (Ag/AgCl reference electrode, Pt wire counter electrode). The Koutecký–Levich (K–L) plots exhibit good linearity. At applied potentials of −0.4, −0.5, and −0.6 V, the calculated electron transfer numbers (n) are 1.88, 2.05, and 2.25, yielding a mean value of 2.05 ± 0.18 (**Figure S16**). These values are close to the theoretical value of 2 for the two‑electron ORR pathway to H_2_O_2_ and are significantly lower than 4, confirming that the competing four‑electron pathway to H_2_O is negligible. These RDE results are consistent with the limited enhancement observed in hole scavenger experiments, collectively demonstrating that 3R-ZnIn_2_S_4_ intrinsically favors the selective 2e^⁻^ ORR for H_2_O_2_₂ generation.


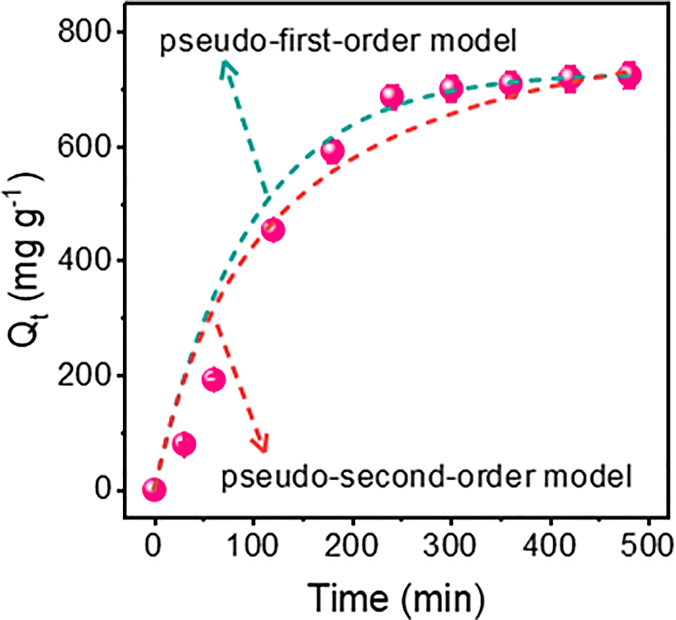


**Figure S18**. Time-dependent adsorption capacity of 3R ZnIn_2_S_4_.

The kinetic data were further analyzed using pseudo-first-order and pseudo-second-order models. Both models described the experimental data reasonably well, with correlation coefficients (R^2^) of 0.975 and 0.962 for the pseudo-first-order and pseudo-second-order models, respectively. The comparable goodness-of-fit suggests that the uranium extraction process involves a combination of physical adsorption and chemisorption mechanisms, consistent with the precipitation of uranyl ions by in-situ generated H_2_O_2_.


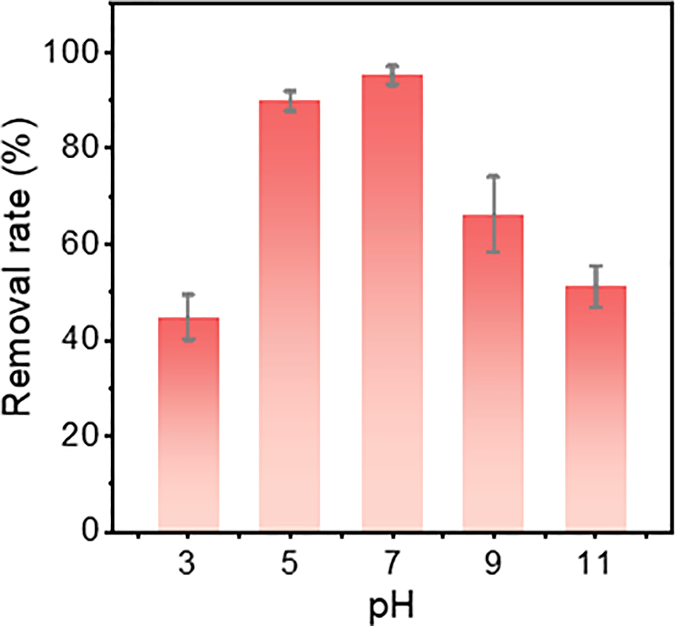


**Figure S19**. Effect of pH on U(VI) removal efficiency by 3R-ZnIn_2_S_4_ over the range of 3–11.


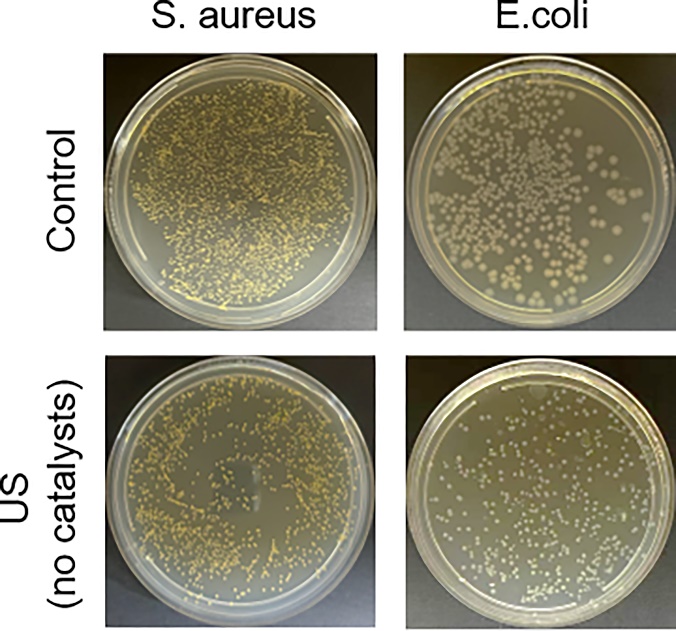


**Figure S20**. The spread plate images before antibacterial treatment.


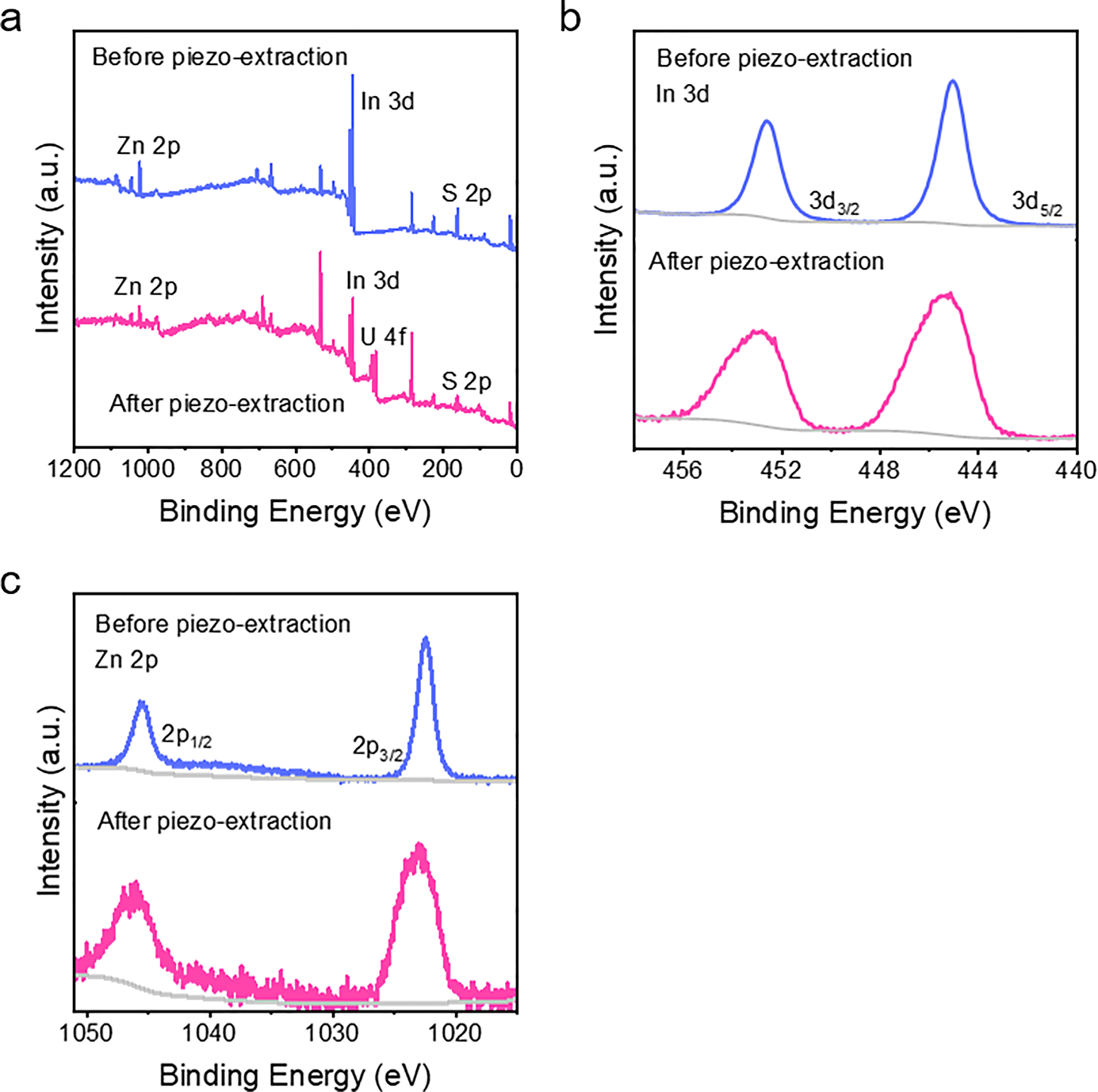


**Figure S21**. (a) Full XPS survey spectrum of 3R ZnIn_2_S_4_ after piezocatalytic uranium extraction, revealing a distinct uranium signal. (b) High-resolution In 3d XPS spectra before and after extraction, showing binding energy shifts indicative of charge redistribution. (c) High-resolution Zn 2p XPS spectra before and after extraction, similarly showing binding energy shifts.

**3.2 Supplementary Tables**

**Table S1.** Calculated total energy (eV) of 3R ZnIn_2_S_4_ under different uniaxial strain conditions (from –6% to +6%) in vacuum and under implicit solvation.

|  | Energy/eV | |
| --- | --- | --- |
| Strain (%) | Vacuum | Solvation |
| -6 | -247.6377 | -248.3688 |
| -4 | -248.7852 | -249.6828 |
| -2 | -249.4005 | -250.4362 |
| 0 | -249.5679 | -250.7007 |
| +2 | -249.3370 | -250.5692 |
| +4 | -248.7664 | -250.0709 |
| +6 | -247.9027 | -249.2628 |

**Table S2.** Mechanism of the piezocatalytic reduction reaction.

| Reactions | steps |
| --- | --- |
| 3R ZnIn_2_S_4_ + ultrasound → e^−^ + h^+^ | (1) |
| U (VI) + 2e^−^ → U (IV) | (2) |
| O_2_ + e^−^ → ⋅O_2_^−^ | (3) |
| U (VI) + ⋅O_2_^−^ → U (IV) + O_2_ | (4) |
| ⋅O_2_^−^ + e⁻ + 2H⁺ → H_2_O_2_ | (5) |
| O_2_ + 2e⁻ + 2H⁺ → H_2_O_2_ | (6) |
| ⋅O_2_^−^ + h⁺ → ^1^O_2_ | (7) |
| ^1^O_2_ + 2e⁻ + 2H⁺ →H_2_O_2_ | (8) |
| UO_2_^2+^ + H_2_O_2_ + 2H_2_O → (UO_2_)O_2_·2H_2_O + 2H⁺ | (9) |
| UO_2_ + 2H_2_O_2_ → (UO_2_)O_2_·2H_2_O | (10) |

**References**

[1] B. Su, S. Wang, W. Xing, et al., “Synergistic Ru Species on Poly(heptazine imide) Enabling Efficient Photocatalytic CO_2_ Reduction with H_2_O beyond 800 nm,” *Angewandte Chemie International Edition* 64 (2025): e202505453, <https://doi.org/10.1002/anie.202505453>.

[2] S. Li, J. Li, Y. Lv, J. Ding, B. Wu, J. Zheng, and H. Zhou, “CeO_2_ nanoparticle-modified BiOI nanoflowers as visible-light-driven heterojunction photocatalyst for tetracycline degradation and antibacterial,” *Materials Today Communications* 41 (2024): 110457, <https://doi.org/10.1016/j.mtcomm.2024.110457>.

[3] Z. Wei, M. Liu, Z. Zhang, W. Yao, H. Tan, and Y. Zhu, “Efficient visible-light-driven selective oxygen reduction to hydrogen peroxide by oxygen-enriched graphitic carbon nitride polymers,” *Energy & Environmental Science* 11 (2018): 2581-2589, <https://doi.org/10.1039/C8EE01316K>.

[4] H. Che, X. Gao, J. Chen, J. Hou, Y. Ao, and P. Wang, “Iodide-Induced Fragmentation of Polymerized Hydrophilic Carbon Nitride for High-Performance Quasi-Homogeneous Photocatalytic H2O2 Production,” *Angewandte Chemie International Edition* 60 (2021): 25546-25550, <https://doi.org/10.1002/anie.202111769>.

[5] J. Li, W. Liu, X. Zhang, P. K. Chu, K. M. C. Cheung, and K. W. K. Yeung, “Temperature-responsive tungsten doped vanadium dioxide thin film starves bacteria to death,” *Materials Today* 22 (2019): 35-49, <https://doi.org/10.1016/j.mattod.2018.04.005>.

[6] G. Kresse, and J. Hafner, “Ab initio molecular dynamics for open-shell transition metals,” *Physical Review B - Condensed Matter* 48 (1993): 13115-13118, <https://doi.org/10.1103/physrevb.48.13115>.

[7] G. F. l. Kresse, J, “Efficient iterative schemes for ab initio total-energy calculations using a plane-wave basis set,” *Physical Review B - Condensed Matter* 54 (1996): 169-185, <https://doi.org/10.1103/PhysRevB.54.11169>.

[8] K. B. John P. Perdew, and Matthias Ernzerhof, “Generalized gradient approximation made simple,” *Phys. Rev. Lett* 77 (1996).

[9] W. Kohn, and L. J. Sham, “Self-Consistent Equations Including Exchange and Correlation Effects,” *Physical Review* 140 (1965): A1133-A1138, <https://doi.org/10.1103/PhysRev.140.A1133>.

[10] S. Grimme, J. Antony, S. Ehrlich, and H. Krieg, “A consistent and accurate ab initio parametrization of density functional dispersion correction (DFT-D) for the 94 elements H-Pu,” *The Journal of Chemical Physics* 132 (2010): 154104, <https://doi.org/10.1063/1.3382344>.

[11] V. Wang, N. Xu, J.-C. Liu, G. Tang, and W.-T. Geng, “VASPKIT: A user-friendly interface facilitating high-throughput computing and analysis using VASP code,” *Computer Physics Communications* 267 (2021): 108033, <https://doi.org/10.1016/j.cpc.2021.108033>.

[12] K. Momma, and F. Izumi, “VESTA: a three-dimensional visualization system for electronic and structural analysis,” *Journal of Applied Crystallography* 41 (2008): 653-658, <https://doi.org/10.1107/s0021889808012016>.

[13] S. Li, J. Li, D. Shi, Z. Sofer, and H. Zhou, “Magnetically driven BiOI/MnFe_2_O_4_ micro/nanorobots for capture, aggregation, and on-site decontamination of biological warfare agents,” *Separation and Purification Technology* 380 (2026): 135351, <https://doi.org/10.1016/j.seppur.2025.135351>.
